# A biological product of *Bacillus amyloliquefaciens* QST713 strain for promoting banana plant growth and modifying rhizosphere soil microbial diversity and community composition

**DOI:** 10.3389/fmicb.2023.1216018

**Published:** 2023-11-02

**Authors:** Libo Tian, Wenlong Zhang, Guang-Dong Zhou, Shu Li, Yongfen Wang, Baoming Yang, Tingting Bai, Huacai Fan, Ping He, Si-Jun Zheng

**Affiliations:** ^1^Yunnan Key Laboratory of Green Prevention and Control of Agricultural Transboundary Pests, Agricultural Environment and Resources Institute, Yunnan Academy of Agricultural Sciences, Kunming, China; ^2^Institute of Tropical and Subtropical Industry Crops, Yunnan Academy of Agricultural Sciences, Baoshan, China; ^3^State Key Laboratory for Conservation and Utilization of Bio-Resources in Yunnan, Ministry of Education Key Laboratory of Agriculture Biodiversity for Plant Disease Management, College of Plant Protection, Yunnan Agricultural University, Kunming, China; ^4^Bioversity International, Kunming, China

**Keywords:** banana, Fusarium wilt of banana, *Bacillus amyloliquefaciens* QST713, microbial diversity and composition, growth promotion

## Abstract

**Introduction:**

Bananas are not only an important food crop for developing countries but also a major trading fruit for tropical and semitropical regions, maintaining a huge trade volume. Fusarium wilt of banana (FWB) caused by *Fusarium oxysporum* f. sp. *cubense* is becoming a serious challenge to the banana industry globally. Biological control has the potential to offer both effective and sustainable measures for this soil-borne disease.

**Methods:**

In order to explore the biocontrol effects of the biological agent *Bacillus amyloliquefaciens* QST713 strain on banana plants, two cultivars, Brazilian and Yunjiao No. 1, with varied resistance to FWB, were used in greenhouse pot experiments.

**Results:**

Results showed that the plant height and pseudostem diameter of banana-susceptible cultivar Brazilian increased by 11.68% and 11.94%, respectively, after QST713 application, while the plant height and pseudostem diameter of resistant cultivar Yunjiao No. 1 increased by 14.87% and 12.51%, respectively. The fresh weight of the two cultivars increased by 20.66% and 36.68%, respectively, indicating that this biological agent has potential effects on plant growth. Analysis of the rhizosphere soil microbial communities of two different cultivars of banana plants showed that TR4 infection and *B. amyloliquefaciens* QST713 strain application significantly affected the bacterial and fungal diversity of Yunjiao No. 1, but not in the cultivar Brazilian. In addition, TR4 infection and QST713 application changed the bacterial community composition of both banana cultivars, and the fungal community composition of Yunjiao No. 1 also changed significantly. Relevance analysis indicated that the relative richness of *Bacillus* and *Pseudomonas* in the rhizosphere of both cultivars increased significantly after QST713 application, which had a good positive correlation with plant height, pseudostem girth, aboveground fresh weight, leaf length, and leaf width.

**Discussion:**

Therefore, the outcome of this study suggests that the biological agent QST713 strain has potential application in banana production for promoting plant growth and modification of soil microbial communities, particularly in the TR4-infected field.

## Introduction

Banana is a perennial monocotyledonous plant and is cultivated in more than 130 countries around the world. Bananas are not only a fruit crop but also a crop with significant global trade income. China has a long cultivation history. A survey by the World Food and Agriculture Organization (FAO) in 2022 found that banana planting in China ranks second in the world (FAO) (Zou and Fan, 2022).

Diseases are significant restrictive factors in banana production, resulting in severe loss of banana yield and quality (Yip, 2017). Fusarium wilt of banana is a soil-borne disease caused by *Fusarium oxysporum* f. sp. *cubense*, (*Foc*). Particularly with the spread of Tropical Race 4 (*Foc* TR4), no banana varieties are immune to its infection, and Fusarium wilt of banana (FWB) has become the most disruptive disease in banana production (Ghag et al., 2015; Li et al., 2019). *Foc* TR4 can be spread in various ways (irrigation water, flowing air, soil, farming tools, etc.), and pathogenic spores can survive in infected soil for over 30 years (Ghag et al., 2015; Li et al., 2019). At present, various control measures have been tested against this disease, such as chemical treatments, disease-resistant varieties, biological control agents, plant quarantine, and cultural controls. Disease resistance breeding is the most effective method, but there is difficulty in implementing this due to the low diversity of genetic background relating to the triploid nature of most commercial banana cultivars. Although crop rotation and intercropping in agricultural production can effectively reduce the damage caused by *Foc* TR4, it is not practical in large-scale implementation (Tao, 2020). The utilization of chemical fertilizers and pesticides can cause environmental issues (Tao, 2020). Therefore, biological control technologies with the characteristics of safety, non-toxicity, and environmental protection have advantages in disease prevention and control (Ploetz, 2006).

Beneficial microorganisms that form mutual interactions with plants are generally considered to have some potential for disease control or development as microbial agents (He et al., 2021). Jayamohan et al. (2020) and Saravanan et al. (2003) found that *Pseudomonas fluorescens* had a high inhibitory activity against Fusarium wilt, and it could also stimulate systemic resistance and raise the ability of bananas to resist diseases. Cheng et al. (2005) also isolated *P. fluorescens* with strong inhibitory activity from banana soil, and further confirmed the inhibitory effect of this bacterium on Fusarium wilt. Fishal et al. (2010) found that the isolated endophytic bacterium UPMP3 (*Pseudomonas* sp.) could induce resistance to Fusarium wilt in susceptible banana varieties, indicating that it is a promising biocontrol agent. Therefore, the development and application of biocontrol agents for diseases have broad prospects (Card et al., 2016; Bubici et al., 2019).

Direct or indirect application of these microorganisms to agricultural production has revealed many successful cases of disease control and/or increase in productivity. Serenade family products are brands of Bayer, based on the *B. amyloliquefaciens* QST713 strain. This strain can produce IAA and 2-3 butanediol to promote plant growth and can quickly colonize the roots, establishing a dense biofilm as a protective barrier. QST713 strain can also activate plant self-defenses to suppress diseases. Studies have shown that 3-indoleacetic acid (IAA) can promote bacterial survival and effective colonization, thereby inhibiting fungal infection to protect plants (Lahlali et al., 2013; Rotolo et al., 2018; Samaras et al., 2021; Clarke et al., 2022). It has been demonstrated that QST713 has played a positive role in promoting cucumber growth, and after application (Bubici et al., 2019), it can resist diseases such as wheat stripe rust (Reiss and Jørgensen, 2017), rice sheath blight (*Rhizoctonia solani*), and leaf spot (*Cochliobolus miyabeanus*) (Harish et al., 2008). However, it is unclear whether QST713 could play the same role in biocontrol and growth promotion of banana plants in the same way as the crops mentioned above.

The application of biological control agents can establish beneficial strains and change the relative dominance of components of the rhizosphere microflora/microbial community, thus reducing pathogen colonization in the banana rhizosphere (Shen et al., 2015; De Vrieze et al., 2018; Bubici et al., 2019). The use of 16S rRNA gene sequencing to evaluate the microbial flora in soils, and thus to find the dominant flora, is a reliable method for studying its suppression of Fusarium wilt in banana (Caporaso et al., 2011). Therefore, QST713 was used to explore the biocontrol effects and growth promotion mechanisms of a biocontrol microorganism in banana plants in this study. We found that the introduction of QST713 not only directly promoted plant growth but also significantly enriched *Pseudomonas* and *Bacillus* in the soil microflora. The aim of this study was to enrich biological growth promoter resources and provide a theoretical foundation for a set of comprehensive measures for sustainable production in the banana industry.

## Materials and methods

### Culture medium and substrate

Potato dextrose agar (PDA) medium (potato 200 g, glucose 15 g, agar 15 g for 1 L) was used for fungal culture and activation. Nutrient agar (NA) medium (yeast extract 5 g, peptone 10 g, sodium chloride 10 g, agar 15 g for 1 L) was used for bacteria culture and activation. MS medium (Coolaber Science & Technology Co., Ltd., Beijing, China) was used for banana tissue culture seedlings propagation. Banana seedling substrate and coconut bran were purchased from Yuxi Letu Technology Co., Yuxi, China.

### Banana cultivars, microbial agents, and pathogens

Resistant cultivar Yunjiao No. 1 (AAA) and susceptible cultivar Brazilian (AAA) propagated by the Agricultural Environmental and Resources Institute, Yunnan Academy of Agricultural Sciences, were used in this study to determine the effects of QST713 on bananas (Zhou et al., 2023). The tissue culture plantlets cultured at 25°C with 16 h/8 h (light/dark) for 20 days were transplanted to the seedling substrate for cultivation. When the banana plantlets had grown with 2–3 more new leaves, they were transplanted into plastic pots (25 cm × 18.3 cm) containing transplanting soil in which the seedling substrate (Banana Planting Substrate, Yunnan Yuxi Leshi Technology Co. Ltd., Yunnan, China) was mixed with fresh soil (native horticultural red soil Ferralosols, which bananas have never been planted in previously) at a ratio of 1:1. One banana plant was planted in each pot. There were 50 banana plantlets in each treatment, and each treatment was replicated three times (Table 1). Routine fertilizer and watering were applied in greenhouse management.

**Table 1:** Treatments of the pot experiments.

Code of treatments	Description
BCK	Control in Brazilian cultivar with normal tap water
BTR4	Brazilian inoculated with TR4 in pot soil
BTR4 + QST713	QST713 drenched to Brazilian and inoculated with TR4 in pot soil
YCK	Control in Yunjiao No. 1 cultivar with normal tap water
YTR4	Yunjiao No. 1 inoculated with TR4 in pot soil
YTR4 + QST713	QST713 drenched to Yunjiao No. 1 and inoculated with TR4 in pot soil

*Bacillus amyloliquefaciens* strain QST713 was provided by Bayer’s Crop Science Department (Leverkusen, Germany). When banana plantlets were transplanted and produced 6 to 7 leaves, a 100 mL QST713 solution (5 mL QST713 original agent labeled as EZU 1631901*1.0 + 95 mL water) was provided to each plant, the roots around the banana plant being drenched. QST713 was applied every 4 weeks, three times in total.

*Foc* TR4 previously isolated by our laboratory in Xishuangbanna, Yunnan Province was used in this experiment (Zhang et al., 2018, 2020). TR4 spore suspension was prepared with 1 × 10^7^ CFU/mL after culturing in PDB medium at 28°C for 48 h. Seven days after the second application of QST713, the soil around each banana plant was drenched with 100 mL TR4 spore suspension (BTR4 + QST713, YTR4 + QST713). The TR4 treatments of Brazilian and Yunjiao No. 1 cultivar was inoculated with TR4 at the same time (BTR4, YTR4). The control treatments of Brazilian and Yunjiao No. 1 cultivar were inoculated with normal tap water (BCK, YCK). There were six different treatments in the experiment, each replicated three times. Each treatment was a total of 150 banana plants, 50 banana plants/treatment/replication (Table 1).

### Investigation on banana growth traits

The banana pot experiment was carried out in the greenhouse of the Agricultural Environment and Resources Institute, Yunnan Academy of Agricultural Sciences. The indoor average illumination time was about 12 h, the average indoor temperature was 32°C (day)/22°C (night), and the average humidity was about 50%–60%. The banana growth traits (plant height, pseudostem girth, leaf number, leaf length, and leaf width) were investigated at 0 day, 28 days, 56 days and 97 days after the first application of QST713. The measurement of plant height was the range from the base of the pseudostem to the intersection of the newest two leaves. Pseudostem girth was measured as the pseudostem diameter 1 cm from the base using vernier calipers. The leaf number was counted as all functional leaves, and the length and width of the first expanded leaf were measured (Fan et al., 2021). After 62 days of TR4 inoculation, the fresh weights of the above and below ground parts of the banana plants were weighed.

### Investigation on disease index of banana

In order to evaluate the efficacy of the QST713 agent for the control of banana wilt in pot experiments, we investigated the incidence on each plant 62 days post-inoculation with TR4 according to the lesion characteristics of bananas. The disease severity index (DSI) standards from Zhang’s et al. (2018) protocol were referred to. DSI was graded from 0 to 4 according to the necrosis degree of the banana corm (“0” represented healthy plants with no internal symptoms and “4” represented severe wilting or that the plant had died).

### Collection of banana rhizosphere soil samples

At 9 weeks (62 days) after inoculation with TR4, the rhizosphere soils of 15 plants of each treatment (CK, TR4, TR4 + QST713) were collected with a sterilized soil shovel. After pulling out the banana plant from the pot and shaking the roots vigorously, soil attached to the surface of the root was regarded as rhizosphere soil and was collected. Before collecting soil samples, tweezers were used to remove residual root system and other sundries from the collected mixed rhizosphere soil. Soil samples were stored at −20°C before DNA extraction. In total, 15-pot-plant soils were sampled, and three pots of soil were mixed into one sample (thus five soil sample replicates) for sequencing. Approximately 20 g rhizosphere soil was collected per plant.

### Soil microbial genomic DNA extraction and detection

Total genomic DNA was extracted from soil samples using the OMEGA Soil DNA Kit (D5625-01) (Omega Bio-Tek, Norcross, GA, United States) following the manufacturer’s instructions, and the isolated DNA was stored at −20°C. The concentration of extracted DNA was determined using a NanoDrop ND-1000 spectrophotometer (Thermo Fisher Scientific, Waltham, MA, United States), and the quality was checked by agarose gel electrophoresis.

### PCR amplification and high-throughput sequencing

PCR amplification of the bacterial 16S rRNA genes V3–V4 region was performed using the forward primer 338F (5′-ACTCCTACGGGAGGCAGCA-3′) and the reverse primer 806R (5′-GGACTACHVGGGTWTCTAAT-3′). PCR amplification of the fungal ITS1 region was performed using the forward primer ITS5F (5′-GGAAGTAAAAGTCGTAACAAGG-3′) and the reverse primer ITS1R (5′-GCTGCGTTCTTCATCGATGC-3′) (Caporaso et al., 2011; Kozich et al., 2013). The PCR contained 5 μL of buffer (5×), 0.25 μL of Fast pfu DNA Polymerase (5 U/μL), 2 μL (2.5 mM) of dNTPs, 1 μL (10 μM) of each forward and reverse primer, 1 μL of DNA template, and 15.75 μL of ddH_2_O. Thermal cycling consisted of initial denaturation at 98°C for 5 min, followed by 25 cycles consisting of denaturation at 98°C for 30 s, annealing at 53°C for 30 s, and extension at 72°C for 45 s, with a final extension of 5 min at 72°C. The amplification products were sent to Personalbio (Personal Biotechnology Co., Shanghai, China) for high-throughput sequencing on the Illumina Miseq platform. Average length of the sequences was 337.77 for 16S rRNA genes, and 269.12 for ITS1 region. QIIME2 2019.4 (Bolyen et al., 2019) was used to performed microbiome bioinformatics. We finally obtained 249,376 ASVs for bacterial communities, and 20,109 ASVs for fungal communities. The sequential data of all samples were loaded into NCBI with accession numbers PRJNA 949124 (16SrRNA) and PRJNA949129 (ITS).

### Data analysis

Kruskal Wallis rank sum test was used to test the difference between Chao1 and Shannon indices. Principal coordinates analysis (PCoA) was used to analyze the visual difference of different community compositions. Permutational MANOVA (PERMANOVA) was used to analyze the differences between community compositions. Co-occurrence patterns were reconstructed by Hmisc package in R and Gephi 0.9.2. Gephi 0.9.2 was used to calculate the modular index. If the Spearman’s correlation coefficient was >0.70 and *p* < 0.05, then co-occurrence was considered robust (Gao et al., 2021). Network stability was measured by the proportion and modularity of negative or positive correlations. The correlation between growth and relative abundance of bacterial genera was calculated by R and visualized with the ggpubr package. Wilcoxon rank sum test was used to analyze the relative abundance of dominant bacteria in the bacterial community between two different treatments. Excel 2019 and IBM SPSS Statistic 25.0 software was used for data processing and statistical analysis, and univariate analysis (LSD and Duncan) in SPSS Statistic 25.0 software was used for variance analysis.

## Results

### Effect of QST713 on promoting banana plant growth

**Figure 1:**
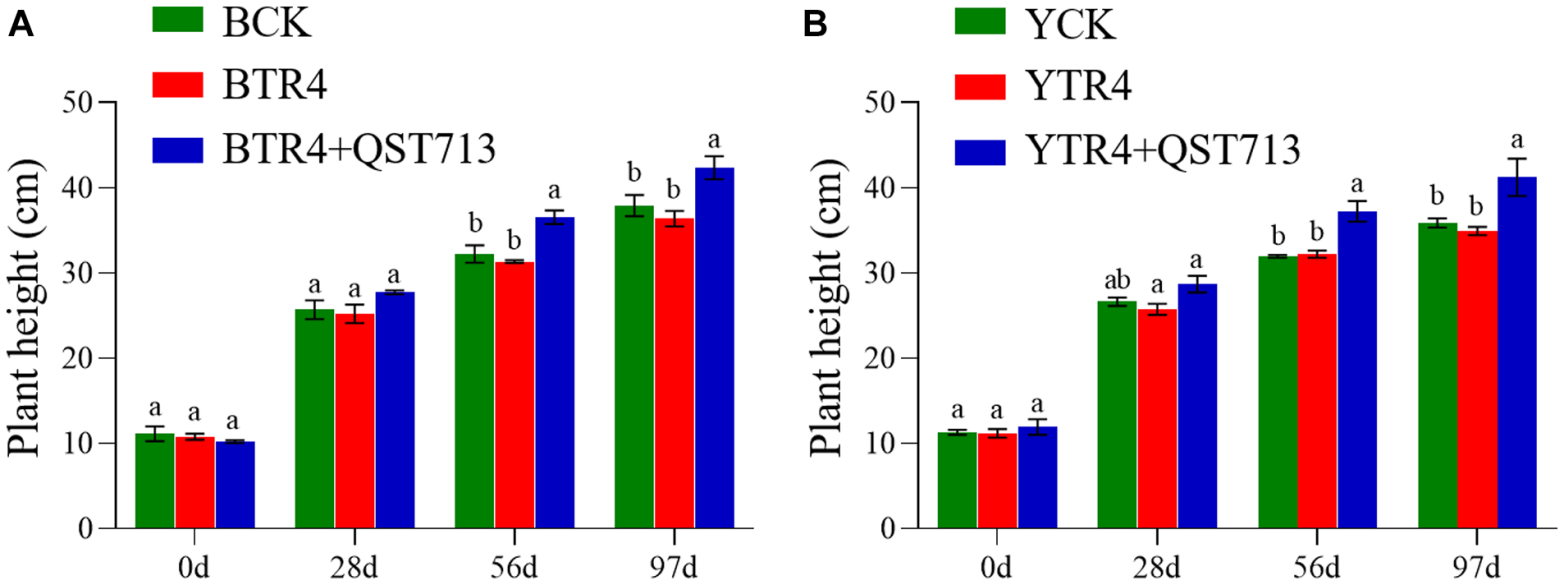
Growth promoting effect of QST713 on banana plant height. **(A)**The plant height of Brazilian. **(B)** The plant height of Yunjiao No. 1. Data are expressed as mean ± standard error. Data for different lowercase letters indicate a significant difference at the 0.05 level.

**Figure 2:**
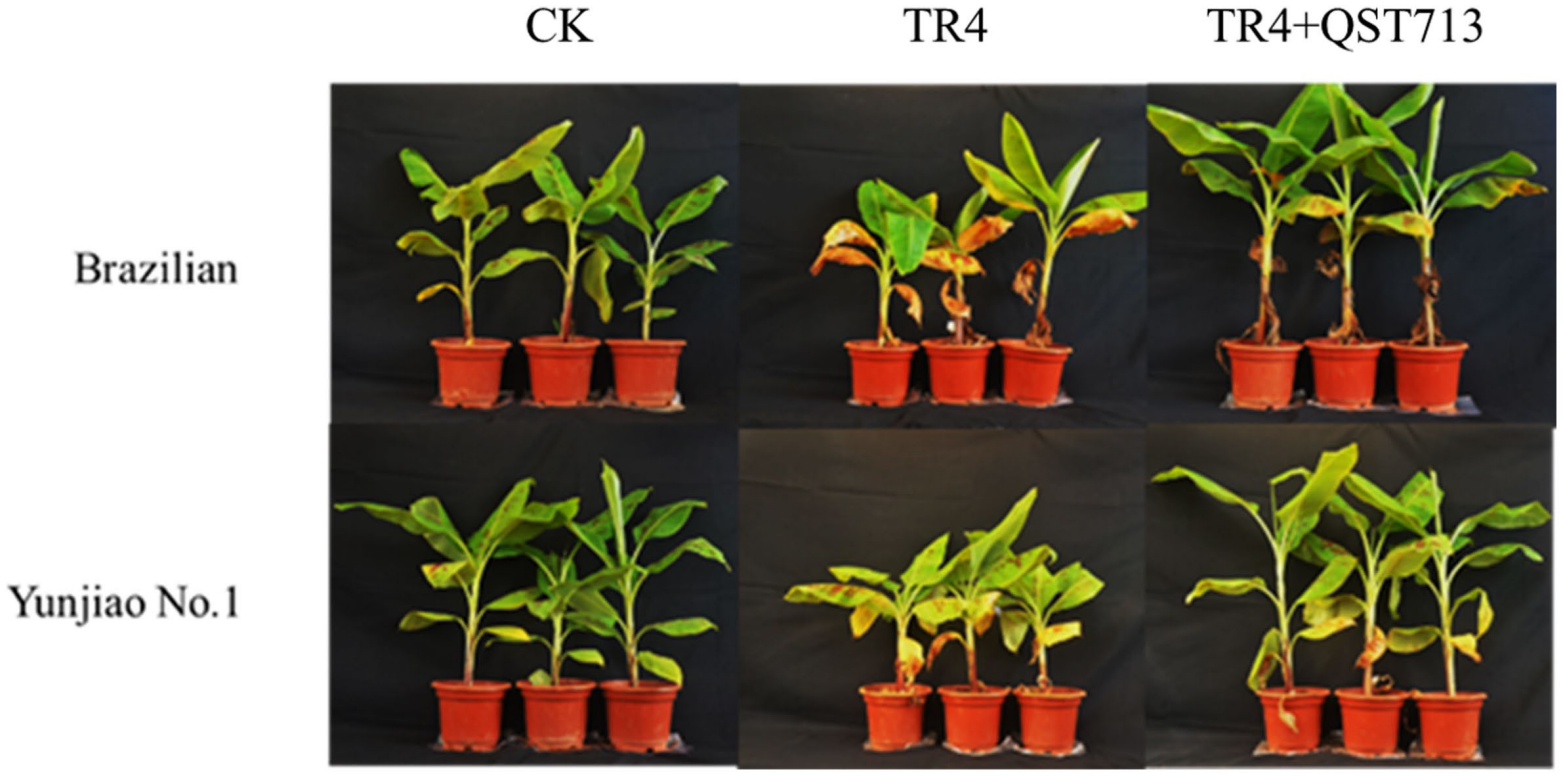
Effect of QST713 on promoting banana plant growth, 97 days after the first application of QST713.

The results of our study showed that the microbial products have a significant effect on promoting plant growth. At 28 days post application (dpa) of QST713 on cultivar Brazilian, there were no significant difference among the CK, TR4, and TR4 + QST713 treatments in plant growth. At 56 dpa, the plant height (36.57 ± 0.79 cm), pseudostem girth (26.48 ± 0.51 mm), leaf length (36.07 ± 0.87 cm), and leaf width (18.00 ± 0.46 cm) of TR4 + QST713 treatments were significantly higher than CK (32.26 ± 1.03 cm, 24.36 ± 0.31 mm, 32.3 ± 1.24 cm, 16.16 ± 0.64 cm) and TR4 (31.36 ± 0.17 cm, 24.07 ± 0.19 mm, 32.69 ± 0.39 cm, 15.67 ± 0.23 cm) treatments. There was no significant difference in leaf number. At 97 dpa, the plant height (42.37 ± 1.36 cm) and pseudostem girth (32.15 ± 0.57 mm) of TR4 + QST713 treatment were significantly higher than the corresponding traits in the TR4 treatment (36.40 ± 0.93 cm, 29.37 ± 0.22 mm) and CK (37.94 ± 1.25 cm, 28.72 ± 1.32 mm), and the leaf length (37.03 ± 1.52 cm) and leaf width (16.82 ± 0.93 cm) were higher than TR4 treatment (32.38 ± 0.98 cm, 13.77 ± 0.06 cm) but not significantly different from the control (36.33 ± 1.65 cm 16.36 ± 0.83 cm) (Figures 1A, 2; Supplementary Figures S1A–S4A).

As for the resistant cultivar Yunjiao No. 1, there was the same trend as Brazilian. Twenty-eight days after the first application of QST713, there were no significant differences in plant growth of QST713 + TR4, TR4, and CK. At 56 dpa, the plant height (37.27 ± 1.22 cm), pseudostem thickness (26.66 ± 0.75 mm), leaf length (39.79 ± 0.87 cm), and leaf width (18.58 ± 0.28 cm) of QST713 + TR4 treatment were significantly higher than those of TR4 treatments (31.97 ± 0.16 cm, 24.79 ± 0.27 mm, 35.43 ± 0.89 cm, 15.86 ± 0.36 cm). At 97 dpa, the plant height (41.24 ± 2.19 cm), pseudostem thickness (33.37 ± 0.52 mm), leaf length (41.5 ± 1.76 cm), and leaf width (17.91 ± 0.67 cm) of the QST713 + TR4 treatment were significantly higher than those of TR4 (34.97 ± 0.46 cm, 29.95 ± 0.41 mm, 34.40 ± 2.47 cm, 15.25 ± 0.39 cm) and the control (35.90 ± 0.53 cm, 29.66 ± 0.51 mm, 38.4 ± 0.34 cm, 15.76 ± 0.25 cm), respectively (Figures 1B, 2; Supplementary Figures S1B–S4B).

**Figure 3:**
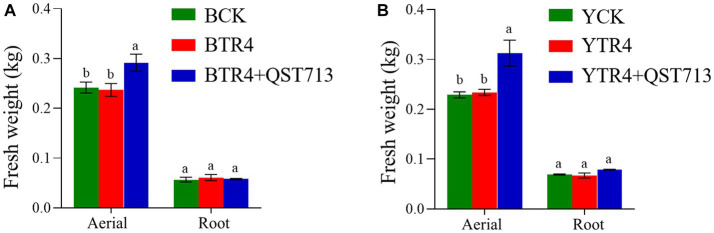
Growth-promoting effects of QST713 on banana plant fresh weight. **(A)** The fresh weight of Brazilian. **(B)** The fresh weight of Yunjiao No. 1. Data are expressed as mean ± standard error. Data for different lowercase letters indicate a significant difference at the 0.05 level.

The fresh weight of aerial biomass in QST713 + TR4 (Brazilian: 0.292 ± 0.017 kg, Yunjiao No. 1: 0.313 ± 0.026 kg) treatment was significantly higher than that of TR4 (Brazilian: 0.237 ± 0.013 kg, Yunjiao No. 1: 0.234 ± 0.006 kg) and the control treatments both in two cultivars. In terms of fresh weight of underground biomass (roots), QST713 + TR4 treatment of Yunjiao No. 1 (0.079 ± 0.001 kg) was significantly higher than that of TR4 (0.067 ± 0.005 kg) but showed no statistically significant difference from CK (0.069 ± 0.001 kg). In this respect, there were also no statistically significant differences between any treatments on cultivar Brazilian (Figure 3).

Disease investigation indicated that QST713 did not decrease the incidence rate of disease of Fusarium wilt of banana. The disease index of BTR4 + QST713 (55.20 ± 3.79) was not significant reduced compared to that of BTR4 (54.17 ± 0.52). The disease index of TTR4 + QST713 (30.73 ± 5.29) was not significant reduced compared to that of TTR4 (26.64 ± 2.61). It was noticed that the disease incidence on Yunjiao No. 1 was prominently lower than Brazilian, which reflects the differences in disease-resistant characteristics of these two cultivars (Supplementary Figures S5, S6).

### Effect of bacterial and fungal community diversity

**Figure 4:**
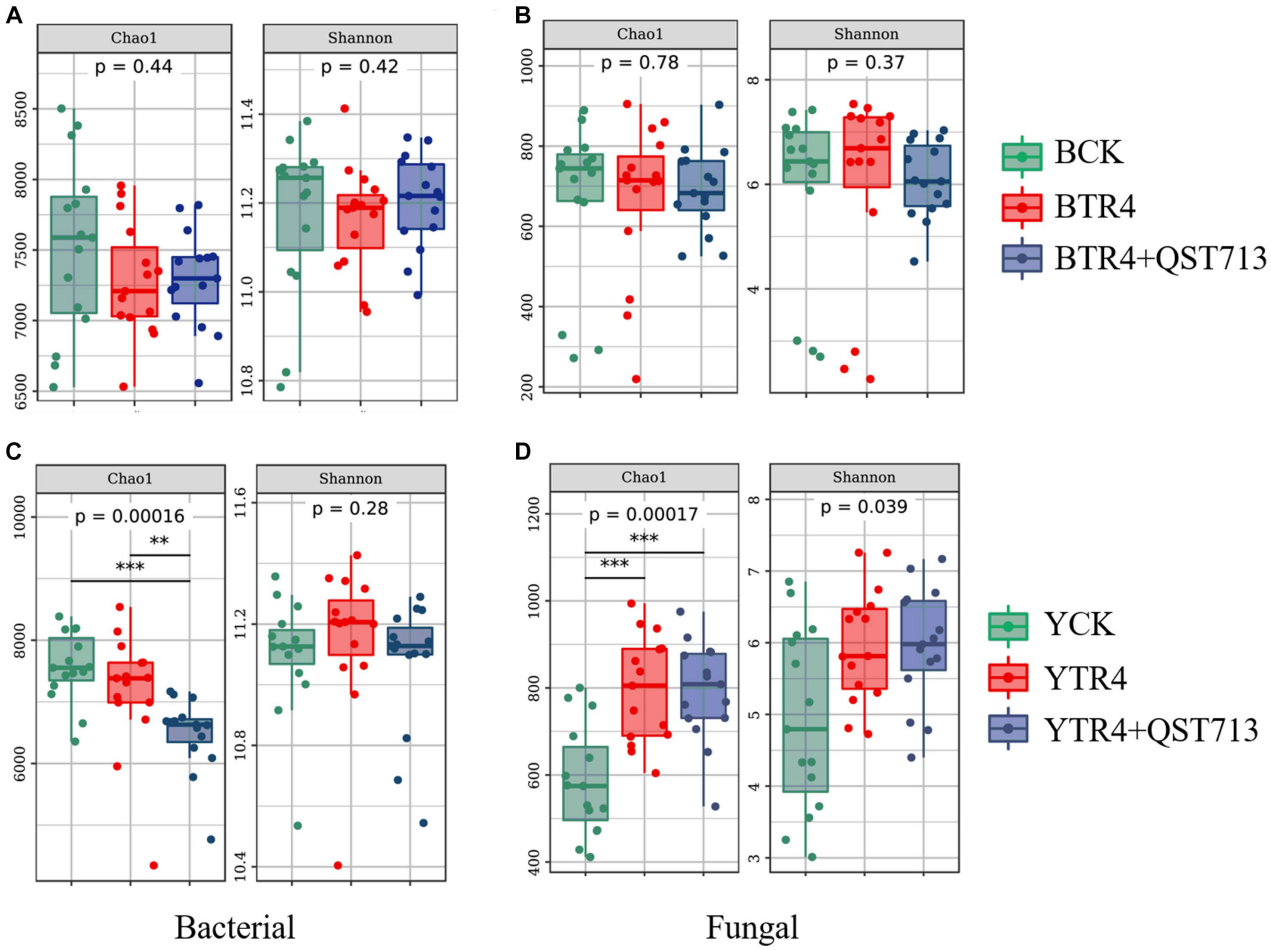
Alpha diversity analysis of microbial bacteria and fungi in banana rhizosphere soil. Alpha diversity in rhizosphere soil bacterial community of Brazilian **(A)** and Yunjiao No. 1 **(C)**. Alpha diversity in rhizosphere soil fungal communities of Brazilian **(B)** and Yunjiao No. 1 **(D)**. BCK represents the control in Brazilian cultivar. BTR4 represents the Brazilian inoculated with TR4. BTR4 + QST713 shows that QST713 was applied to Brazilian and inoculated with TR4. YCK represents the control in Yunjiao No. 1 cultivar. YTR4 represents Yunjiao No. 1 inoculated with TR4. YTR4 + QST713 represents QST713 applied to Yunjiao No. 1 and inoculated with TR4.

In order to determine the effect of QST713 on the soil microbial diversity (Chao1 and Shannon index) of two different banana cultivars (Brazilian and Yunjiao No. 1), we compared the alpha diversity of bacterial and fungal communities in rhizosphere soils. The results showed that there were no statistically significant differences in bacterial community richness (Chao1 index) between BCK, BTR4, and BTR4 + QST713 treatments in the Brazilian cultivar (Figure 4A and Supplementary Table S1). However, there was a statistical significance in bacterial community richness from YTR4 to YTR4 + QST713 treatments in Yunjiao No. 1 (Figure 4C and Supplementary Table S1). The Chao1 index of YTR4 + QST713 was significantly lower than YTR4. In addition, the Chao1 index of YTR4 + QST713 was significantly lower than that of YCK treatment (Figure 4C and Supplementary Table S1). However, there was no statistical significance in the diversity of the bacterial communities in either cultivar.

From the perspective of alpha diversity of fungal community, there was no significant difference in fungal community richness between BCK, BTR4, and BTR4 + QST713 in Brazilian cultivar (Figure 4B and Supplementary Table S1). However, in Yunjiao No. 1, compared with YCK treatment, YTR4 + QST713 and YTR4 treatments showed significantly higher fungal community richness (Chao1 index) (Figure 4D and Supplementary Table S1).

The results showed that there were no statistically significant differences in bacterial and fungal community diversity and richness (Chao1 and Shannon index) between BCK, BTR4, and BTR4 + QST713 treatments, and there were also no statistically significant differences in bacterial and fungal community diversity (Shannon index) between YCK, YTR4, and YTR4 + QST713 treatments. However, there were significantly differences in the richness of bacterial and fungal communities (Chao1 index) between YTR4 and YTR4 + QST713 treatments. The Chao1 index of YTR4 + QST713 was significantly lower than YTR4 and YCK in the bacterial community, and the Chao1 index of YCK was dramatically lower than YTR4 + QST713 and YTR4 in the fungal community.

### Effect of bacterial and fungal community composition

**Figure 5:**
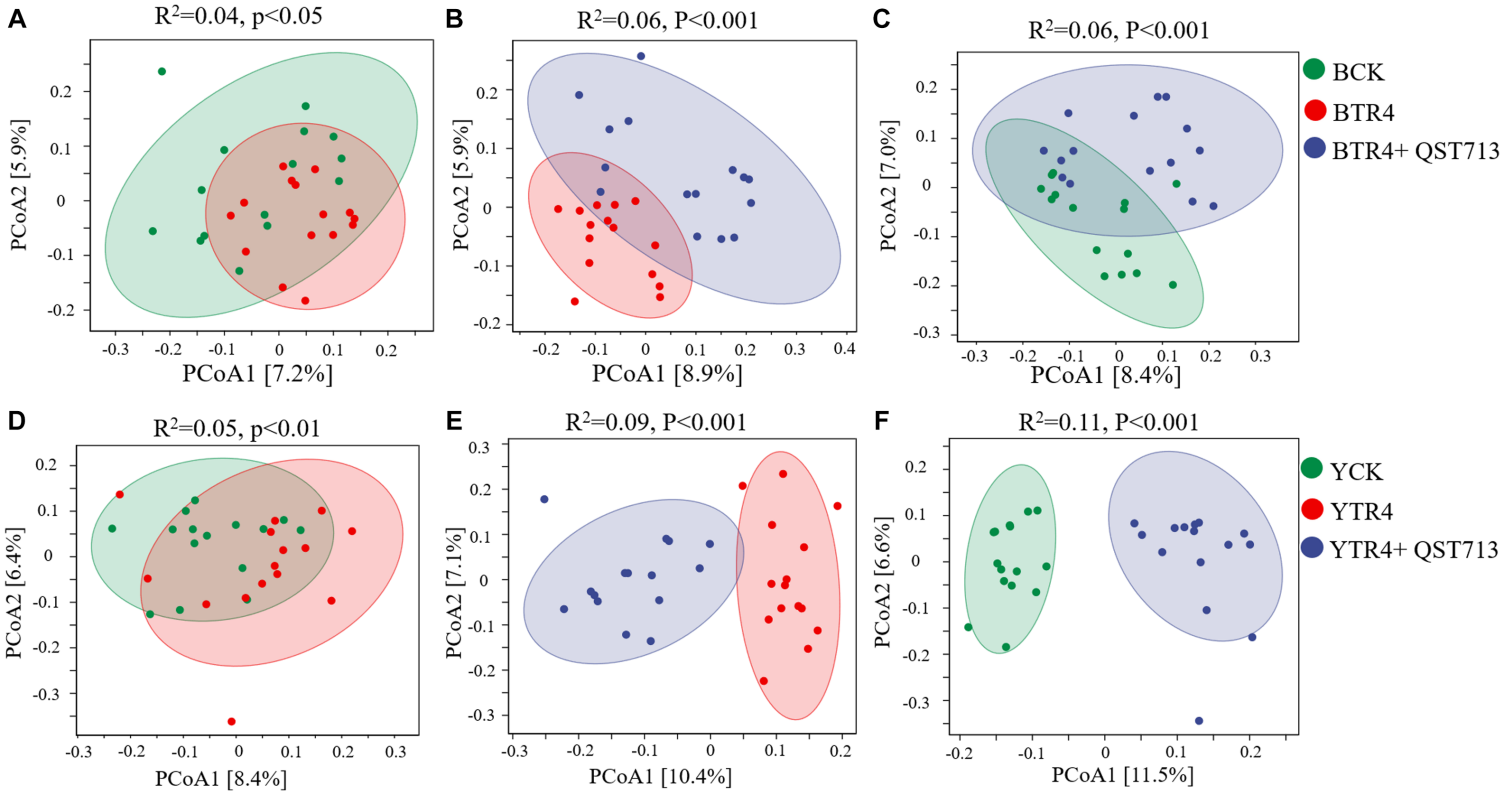
Principal coordinate analysis (PCoA) of bacterial community structure based on the Bray–Curtis distance metric among all soil samples. **(A)** Difference between CK vs. TR4 in bacterial communities’ composition of Brazilian’s soil rhizosphere. **(B)** Difference between TR4 vs. TR4 + QST713 in bacterial communities’ composition of Brazilian’s soil rhizosphere. **(C)** Difference between CK vs. TR4 + QST713 in bacterial communities’ composition of Brazilian’s soil rhizosphere. **(D)** Difference between CK vs. TR4 in bacterial communities’ composition in Yunjiao No. 1’s soil rhizosphere. **(E)** Difference between TR4 vs. TR4 + QST713 in bacterial communities’ composition in Yunjiao No. 1’s soil rhizosphere. **(F)** Difference between CK vs. TR4 + QST713 in bacterial communities’ composition in Yunjiao No. 1’s soil rhizosphere. *R*^2^ represents differential explanation rate, *P* represents significance by Permutational MANOVA. Percentages in parentheses represent the variance explained by the respective axis.

PCoA and PERMANOVA were used to analyze the effects of QST713 application and pathogen TR4 on the bacterial and fungal communities of banana rhizosphere microbial. From the perspective of bacterial community composition in Brazilian, compared with BCK treatment, the composition of bacterial communities treated with BTR4 was statistically significant (*R*^2^ = 0.04, *p* < 0.05) (Figure 5A). In addition, there were significant differences between BTR4 + QST713 and BCK treatments in bacterial communities (*R*^2^ = 0.06, *p* < 0.001) (Figure 5C). BTR4 + QST713 treatment was also significantly different from BTR4 treatment (*R*^2^ = 0.06, *p* < 0.001) (Figure 5B). Therefore, the application of QST713 changed the composition of the bacterial community additionally to the changes brought about by TR4 alone. These changes had the same tendency in resistant cultivar Yunjiao No. 1 (Figures 5D–F). The results showed that the application of QST713 and inoculation of TR4 could significantly change the rhizosphere soil bacterial community of banana plants.

**Figure 6:**
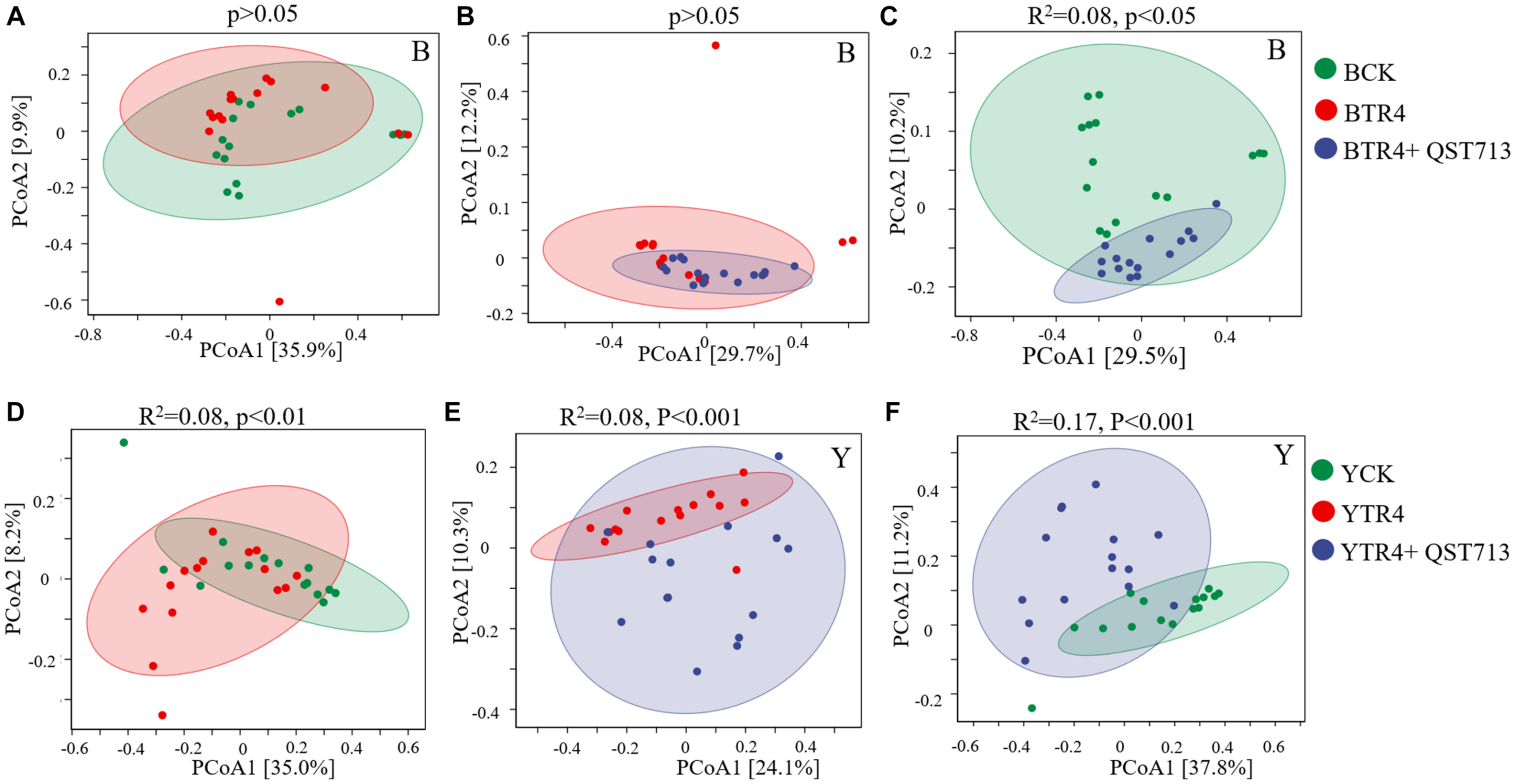
Fungal communities’ composition of banana soil rhizosphere. **(A)** Difference between CK vs. TR4 in fungal communities’ composition in Brazilian’s soil rhizosphere. **(B)** Difference between TR4 vs. TR4 + QST713 in fungal communities’ composition in Brazilian’s soil rhizosphere. **(C)** Difference between CK vs. TR4 + QST713 in fungal communities’ composition in Brazilian’s soil rhizosphere. **(D)** Difference between CK vs. TR4 in fungal communities’ composition in Yunjiao No. 1’s soil rhizosphere. **(E)** Difference between TR4 vs. TR4 + QST713 in fungal communities’ composition in Yunjiao No. 1’s soil rhizosphere. **(F)** Difference between CK vs. TR4 + QST713 in fungal communities’ composition in Yunjiao No. 1’s soil rhizosphere. *R*^2^ represents differential explanation rate by Permutational MANOVA, *P* represents significance. Percentages in parentheses represent the variance explained by the respective axis.

From the point of view of fungal community composition, there were no statistically significant differences between the compositions of fungal communities for the Brazilian BTR4 vs. BCK treatments (Figure 6A, *p* > 0.05) or BTR4 vs. BTR4 + QST713 treatments (Figure 6B, *p* > 0.05). Compared with BCK treatment, there was a significant difference in BTR4 + QST713 treatment in the composition of fungal communities (Figure 6C, *R*^2^ = 0.08, *p* < 0.05). As for Yunjiao No. 1, there were significant differences between YTR4 vs. YCK (Figure 6D, *R*^2^ = 0.08, *p* < 0.01), YTR4 vs. YTR4 + QST713 (Figure 6E, *R*^2^ = 0.08, *p* < 0.001), and YTR4 + QST713 vs. YCK (Figure 6F, *R*^2^ = 0.17, *p* < 0.001). It can be seen that TR4 infection and QST713 application could both change the bacterial community composition of two banana cultivars, and the fungal community composition of Yunjiao No. 1 also changed significantly.

### Effect of QST713 on maintaining the stability of rhizosphere soil microorganism community composition

**Figure 7:**
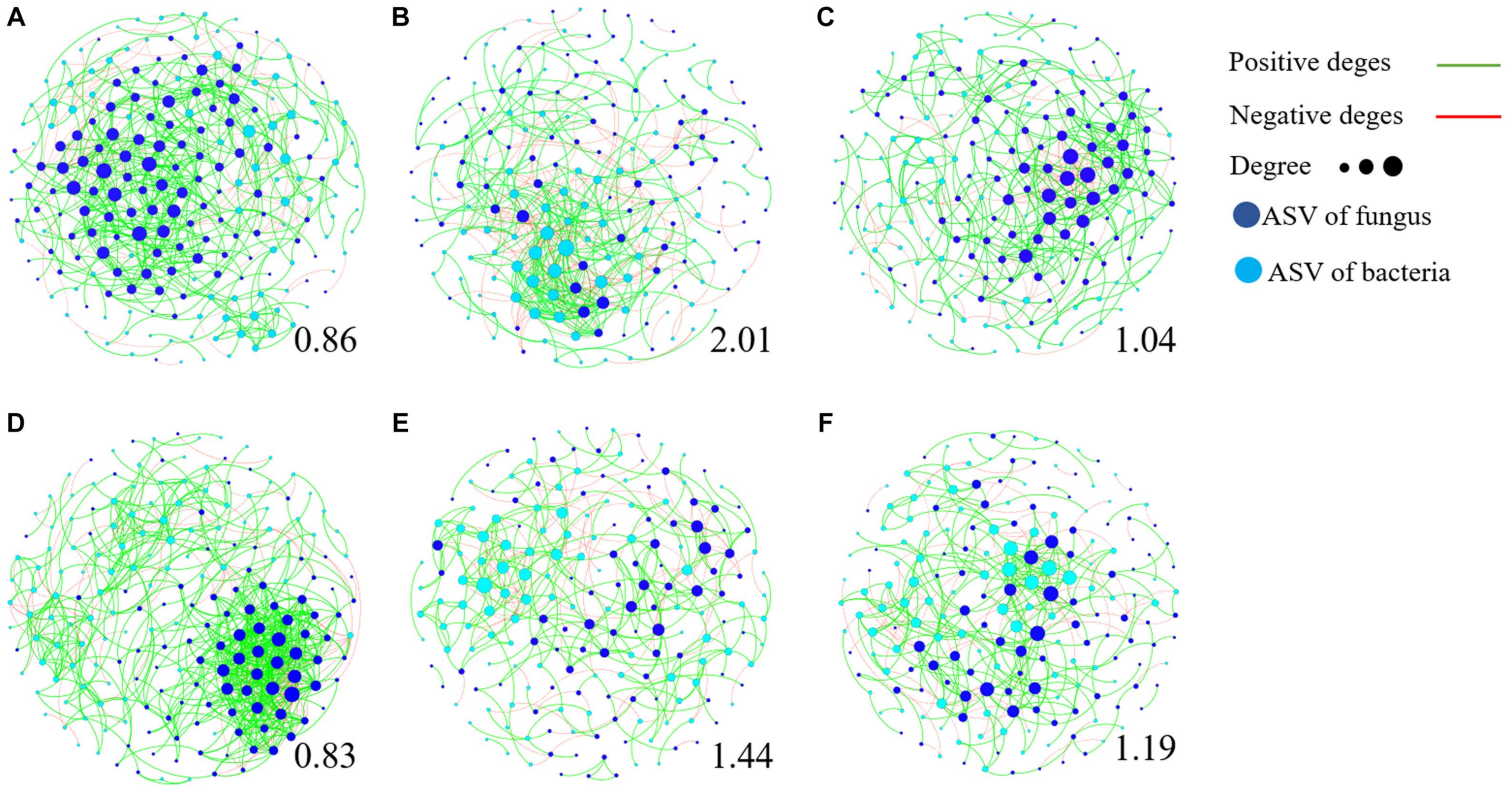
Transboundary network diagram of dominant amplicon sequence variant (ASV, relative abundance >0.1%). The dots represent ASVs, and the lines represent the relationship between ASVs. The red edges are negatively correlated, while the green edges are positively correlated. The dark blue dots are fungal ASV, while the light blue dots are bacterial ASV. The larger the dots are, the more ASV is associated with them, and the greater degree of connected edges. The number below each graph represents its modularity index. The higher the modularity index, the more stable the community structure. **(A)** BCK. **(B)** BTR4. **(C)** BTR4 + QST713. **(D)** YCK. **(E)** YTR4. **(F)** YTR4 + QST713.

We further used the negative correlation edges and modularity index to determine the stability of community network relationships (Figure 7; Supplementary Figure S7). The higher the ratio of negative correlation edges and modularity index, the more stable the community structure. The results showed that the ratio of modularity index and negative correlation edge of TR4 inoculation (Figures 7B,E) were significantly higher than that of CK treatment (Figures 7A,D), while the influence of TR4 + QST713 treatment lay between them (Figures 7C,F). This shows that ST713 has a significant effect on maintaining the stability of rhizosphere soil microorganism community composition in the presence of TR4.

### Effect of QST713 on the specific genus of the rhizosphere soil microorganism community

**Figure 8:**
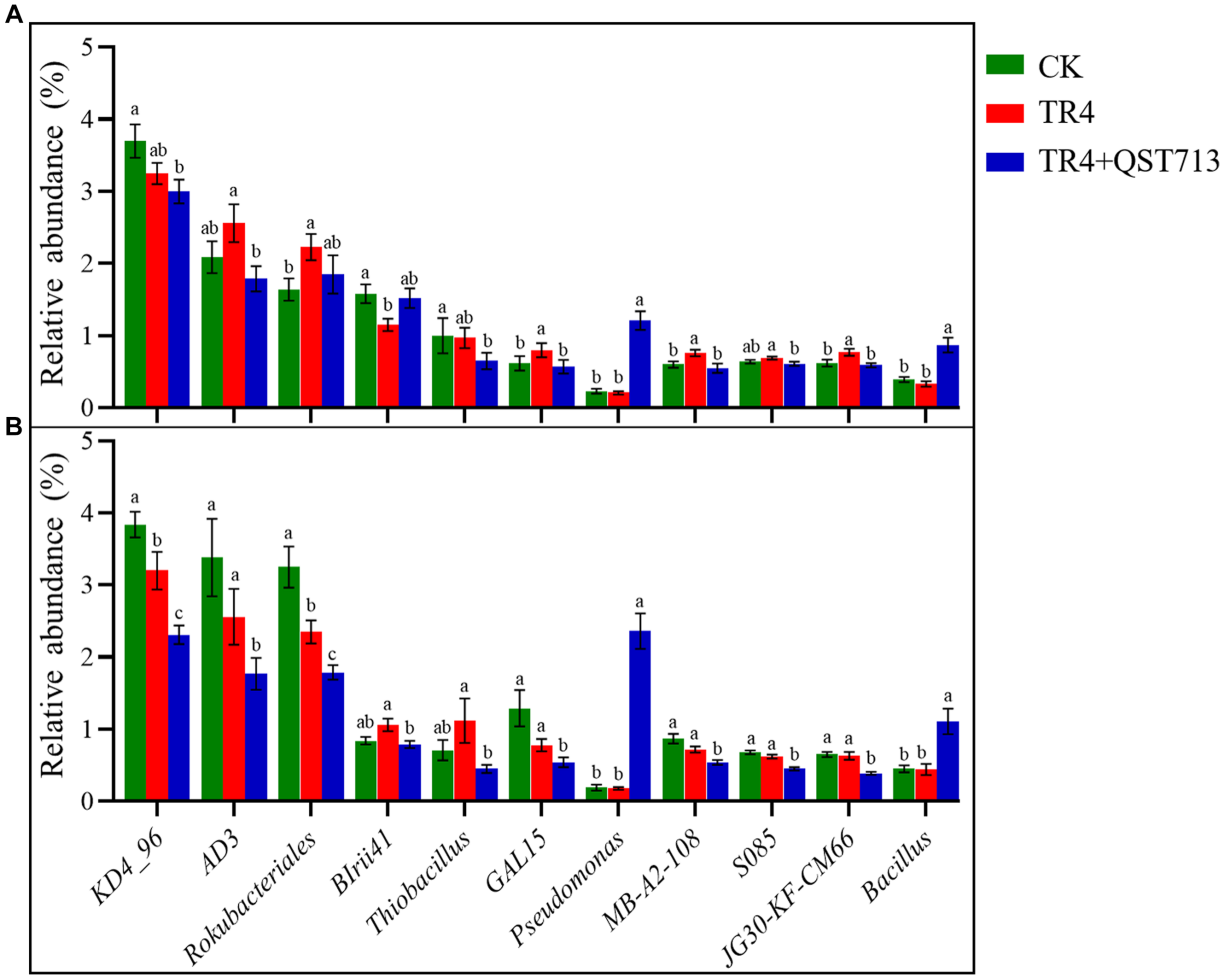
Significantly dominant bacterial genera (relative abundance >0.5%). **(A)** Significant genus of bacteria in banana rhizosphere in Brazilian cultivar. **(B)** Significant genus of bacteria in banana rhizosphere in Yunjiao No. 1 cultivar. Data with different lowercase letters indicate a significant difference at the 0.05 level.

The above diversity analysis showed that the bacterial community composition was significantly different in different treatments, but the fungal communities were significantly different between the two cultivars. The change of bacterial community could be the potential reason for the growth of the two cultivars. Therefore, Wilcoxon rank sum test of variance was used to analyze the relative abundance of dominant bacteria in the bacterial community between different treatments of the two cultivars, so as to identify the dominant bacteria with specific changes. The outcome of this study indicated that compared to the TR4 treatments, the bacteria of *Pseudomonas* and *Bacillus* were significantly up-regulated in TR4 + QST713 treatment (Figure 8), which may provide a key insight into the observed growth promotion effect on banana plants.

### Linking the dominant bacteria with banana plant growth

**Figure 9:**
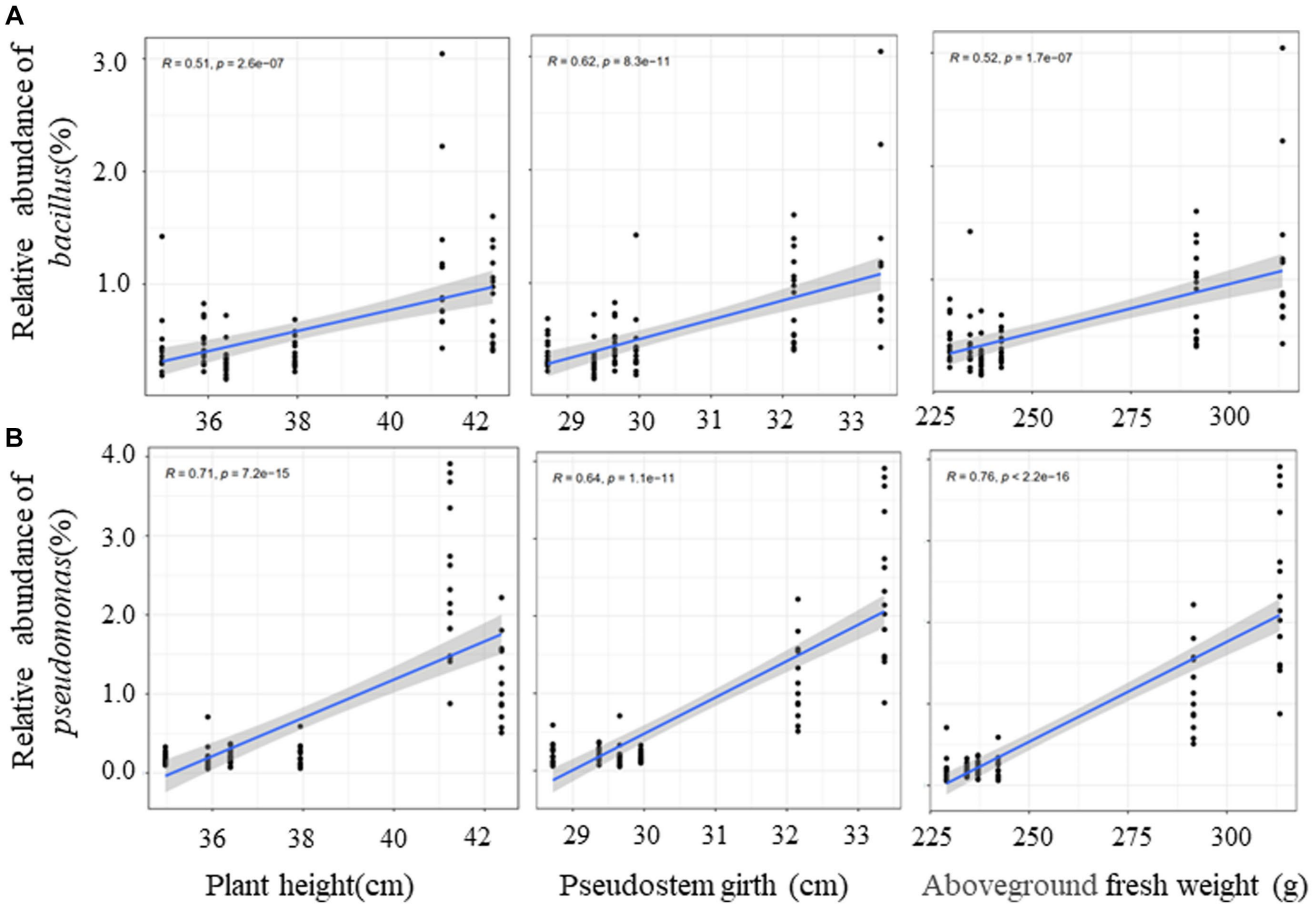
Correlation between dominant bacteria and growth traits of banana based on Spearman. The *y*-axis indicates the relative abundance of dominant bacteria, and the *x*-axis indicates the agronomic traits. Blue lines show linear regression. *R* represents differential explanation rate, *P* represents significance.

In order to clarify the correlation between dominant bacteria (*Pseudomonas* and *Bacillus*) and plant growth effect, the correlation analysis of the relative abundance of dominant bacteria and banana growth parameters were conducted. The outcome of this study indicated that that the relative abundance of the two genera had a significant positive correlation with plant height, pseudostem girth, and above-ground fresh weight (Figure 9). It was the same for leaf length and leaf width but had no correlation with leaf number (Supplementary Figure S8). There was a low positive correlation between *Bacillus* and underground fresh weight (Supplementary Figure S8). Finally, we conclude that QST713 can significantly promote the growth of banana plants by altering the microbial community of bananas, especially by increasing the relative abundance of *Pseudomonas* and *Bacillus*.

## Discussion

Plant rhizosphere growth promoting bacteria (PGPR) is an important component of biocontrol factors, which can effectively promote plant growth (Hu et al., 2004). Zheng et al. (2013) found that the application of biocontrol agents could significantly increase the number of plant roots and improve the microbial diversity of rhizosphere soil. Dan et al. (2018) showed that biocontrol bacteria can effectively promote the growth of melon seedlings and can significantly resist the growth of the pathogen of melon vine blight. *B. amyloliquefaciens* W19 was found to observably decrease the incidence of FWB and promote the growth of banana plants (Wang et al., 2013). Our study showed that the biocontrol agent QST713 could significantly promote the growth of bananas and has a significant effect on plant height, pseudostem girth, fresh weight, and other traits of bananas (Figures 1–3; Supplementary Figures S1–S4). However, through the symptom observation of banana corms, we found that QST713 had no clear control effect on TR4 under the current test conditions although there were clear differences in the level of resistance between the two cultivars, which was confirmed by Zhou et al. (2023) (Supplementary Figures S5, S6). Why did QST713 not suppress the disease in the current study? This could be due to the low dosage application of QST713 in this study. Why QST713 significantly promoted banana growth while there was no clear TR4 suppression in the current dosage application needs further investigation, with various dosage level applications, in the next step of our research. QST713 has also been shown to significantly induce disease resistance in canola, bean, grape, mushroom, and rice (Lahlali et al., 2013; Rotolo et al., 2018; Samaras et al., 2021; Clarke et al., 2022). The application of QST713 improved the “tolerance” of disease in bananas as evidenced by the better growth of leaves and pseudostems (Supplementary Figures S1–S4). Unfortunately, we did not find that QST713 had significant disease resistance against Fusarium wilt in banana with the applied dosage, and we need to monitor its performance at different dosage levels in banana plants in the next step of our research. We also need field trials to further study open field conditions. For the effect caused by QST713 application that triggered the dynamics of soil microorganisms, we measured the soil microbial community changes in bananas.

The soil microbial community is the most important functional component of soil biota. Microorganisms not only provide nutrients for the soil but also help plants adapt to the environment. They respond quickly to changes in soil microecological environment and stress, resulting in changes in microbial diversity and community structure (Chen et al., 2019). The role of biocontrol agents in field soil is greatly affected by the soil environment. It is of great important that new and effective biocontrol resources from different habitats are obtained for the control of Fusarium wilt in banana. There are interactions, such as antagonism, promotion, and coexistence, between soil microorganisms. Adding specific microorganisms to soil artificially would cause significant changes to the soil microbial community (Fan et al., 2021).

In this study, 16S rRNA of bacteria and ITS of fungi were sequenced through the Illumina platform to quantitatively analyze the diversity of soil bacterial and fungal communities. The results showed that the biocontrol agent (QST713 ASO) increased the relative abundance of certain microorganisms in soil bacterial and fungal communities, and significantly increased/the diversity of fungal communities, indicating that the application of this biocontrol agent led to the increase of soil bacterial and fungal species, and improved the diversity of bacterial and fungal communities (Figures 4–6). Notably, the application of QST713 had varying effects on soil microorganisms depending on the banana cultivar. It can be seen that for the susceptible cultivar Brazilian, the inoculation/application of pathogen or QST713 did not affect the abundance and diversity of rhizosphere soil microorganisms (Figure 4), which may be related to its susceptible characteristics. After the application of QST713, the variation of microbial diversity of Brazilian rhizosphere was more stable (Figure 7). For the resistant cultivar Yunjiao No. 1 (Zhou et al., 2023), the inoculation of pathogens or QST713 application reduced the abundance of bacteria but increased the abundance of fungi (Figure 4), indicating that Yunjiao No. 1 somehow reacted more in the presence of a fungal pathogen, then influenced other fungi in the rhizosphere.

After application of the biocontrol agent, QST713, the composition of the soil microbial community changed significantly. The PERMANOVA analysis results showed that the bacterial and fungal communities of the control treatment were significantly different from those of the biocontrol agent QST713 application, indicating that the difference in soil microbial community structure might be one of the main factors regulating the banana plant growth. In addition, the PERMANOVA analysis results showed that the effect of inoculation of pathogens or QST713 application on the bacterial and fungal communities of resistant cultivar Yunjiao No. 1 is always greater than that on the susceptible cultivar Brazilian. This shows that the response of the microbiota of disease-resistant cultivars to interference is higher than that of susceptible cultivars, which indicates that disease-resistant cultivars can effectively adjust the response of the microbiota in the face of environmental changes. Our results clearly demonstrated that there is a significant interaction between host cultivar and pathogen inoculation or biocontrol agent application. Both cultivars showed the same trend in co-occurrence networks, possibly because TR4 inoculation disturbed the balance of the rhizosphere microbiome of the banana, and an increased microbe-mediated immune response disrupted the microenvironment required for normal growth. However, QST713 could reduce the disturbance caused by TR4 inoculation without changing the occurrence of disease and maintain a microbiome that was more conducive to the normal growth of banana.

The data from this study also indicate that after the application of QST713, the relative abundance of *Pseudomonas* and *Bacillus* increased significantly, and their abundance was positively correlated with the growth characteristics of bananas, indicating that the microbial abundance of these two genera (*Pseudomonas* and *Bacillus*) could be directly (or indirectly) connected to the observed enhanced growth of banana plants. Due to the fixed amount of QST713 for plant application and *Foc* TR4 for plant inoculation in this study, we did not quantify *Foc* TR4 and QST713 in pot soil. Therefore, we cannot analyze the correlation between *Foc* TR4 and *Pseudomonas* and *Bacillus*. However, *Pseudomonas* is widely found in nature and is an important part of soil micro-ecosystems. It participates in important metabolic activities such as carbon and nitrogen cycling in the environment and can biodegrade some environmental pollutants (Meng et al., 2022). Studies have shown that *Pseudomonas* species/strains can synthesize secondary metabolites such as siderophores and IAA to provide mineral nutrients for plants and regulate plant growth (Wang et al., 2012; Qian et al., 2022). Some researchers have also shown that it can induce systemic resistance and effective root colonization in plants (Luo, 2016; Qu et al., 2020; Tang, 2020). *Bacillus* species are also a very important group of growth-promoting bacteria. We have isolated and identified one *Bacillus* strain, YN 1910, from banana-suppressive soil. *In vitro* and pot experiments from our study showed that this strain significantly suppressed pathogen and TR4 control effects (78.43%–81.76%) and also had a significant growth promotion effect on banana plants (Fan et al., 2023). At present, a number of “biological” products with a type of *Bacillus* as the main active ingredient have been commercialized. *B. amyloliquefaciens* products can be used as fungicides and plant growth promoters to control plant diseases and promote growth through root treatment. Auxin and similar metabolites such as cytokinin, zeatin, and abscisic acid have also been found in liquid culture (Idris et al., 2004). *B. amyloliquefaciens* BEB33 isolated by Yang et al. (2014) showed good biocontrol effect on wilt, and the strain could produce IAA and siderophore, which could promote the growth of banana plants and showed high biocontrol potential. In addition, we noticed that the relative abundance of *KD4*-*96* of the two cultivars decreased after inoculation with QST713. This kind of bacteria belongs to the *Chloroflexi*, which was reported to be positively correlated with plant growth (Chen et al., 2021), but there was little change compared with *Pseudomonas* and *Bacillus*.

The literature indicates that microbial species that normally promote plant growth may also play a role in plant disease resistance. In this study, the rhizosphere microbial diversity of banana soil was determined to identify species that changed significantly after the application of QST713. These species are considered important to biological control practice. This study also explained why QST713 can increase the tolerance of bananas to disease. QST713 not only directly promotes plant growth but also accumulates several beneficial microorganisms that may help in the long-term management of pathogenic bacteria or fungi. The mechanism behind this could be that the introduction of bacteria into the soil as key community members stimulates other potential growth-promoting species in indigenous beneficial microorganisms. These complex microbial communities could play a decisive role in promoting plant growth and health.

## Conclusion

This study showed that the biocontrol agent *Bacillus amyloliquefaciens* QST713 strain could significantly promote the growth of banana in terms of plant height, pseudostem girth, and leaf traits of banana. Soil microbial diversity sequencing showed that the biocontrol agent *Bacillus amyloliquefaciens* QST713 could also significantly change the composition of the soil microbial community and significantly modify the diversity of the soil fungal community. Furthermore, the application of this biocontrol agent can significantly increase the abundance of *Pseudomonas* and *Bacillus*, and the abundance of these two genera is significantly and positively correlated with banana growth. The effect of QST713 on TR4 suppression with different applied dosages should be investigated, and we need to monitor its performance in banana plants with the application of different dosage levels in the next step of our research.

## Data availability statement

The datasets presented in this study can be found in online repositories. The names of the repository/repositories and accession number(s) can be found in the article/Supplementary material.

## Author contributions

LT and PH: conceived, designed and performed the experiment, analyzed the data, and prepared the manuscript. S-JZ: conceived and designed the experiment, prepared and edited the manuscript, and supervised the research project and provided funding support. WZ, G-DZ, SL, YW, BY, TB, and HF: performed the experiment and analyzed the data. All authors contributed to the article and approved the submitted version.
