# Supplementary material for: A biological product of Bacillus amyloliquefaciens QST713 strain for promoting banana plant growth and modifying rhizosphere soil microbial diversity and community composition
Source: Front Microbiol. 2023 Nov 2;14:1216018. doi: 10.3389/fmicb.2023.1216018 (PMC10653307; doi:10.3389/fmicb.2023.1216018)
Supplement: Supplementary file 1 [file Presentation_1.pdf]

A biological product of *Bacillus amyloliquefaciens* QST713 strain for promoting banana plant growth and modifying rhizosphere soil microbial diversity and community composition

Libo Tian[1], Wenlong Zhang[1], Guang-Dong Zhou[1], Shu Li[1], Yongfen Wang[1,3], Baoming Yang[1], Tingting Bai[1], Huacai Fan[1], Ping He[1,2*], Si-Jun Zheng[1,4*]

[1]Yunnan Key Laboratory of Green Prevention and Control of Agricultural Transboundary Pests, Agricultural Environment and Resources Institute, Yunnan Academy of Agricultural Sciences, Beijing Road 2238, Kunming 650205, Yunnan, China
[2]State Key Laboratory for Conservation and Utilization of Bio-Resources in Yunnan, Ministry of Education Key Laboratory of Agriculture Biodiversity for Plant Disease Management, College of Plant Protection, Yunnan Agricultural University, Kunming 650201, Yunnan, China
[3]Institute of Tropical and Subtropical Industry Crops, Yunnan Academy of Agricultural Sciences, Baoshan, China
[4]Bioversity International, Beijing Road 2238, Kunming 650205, Yunnan, China

*** Correspondence:**
Corresponding Author

Sijun Zheng: sijunzheng63@163.com;

Ping He: heping_superv@163.com.

Supplementary materials

Supplementary Table S1. Differential analysis of alpha diversity between bacterial and fungal communities under different treatments by Kruskal Wallis rank sum test

Community	Cultivars	Index	Df	F	P
Bacterial	Brazilian	Chao1	2	1.203	0.44
		Shannon	2	0.3	0.42
	Yunjiao No.1	Chao1	2	7.931	0
		Shannon	2	0.603	0.28
Fungal	Brazilian	Chao1	2	0.29	0.78
		Shannon	2	0.228	0.37
	Yunjiao No.1	Chao1	2	15.118	0
		Shannon	2	6.703	0.04

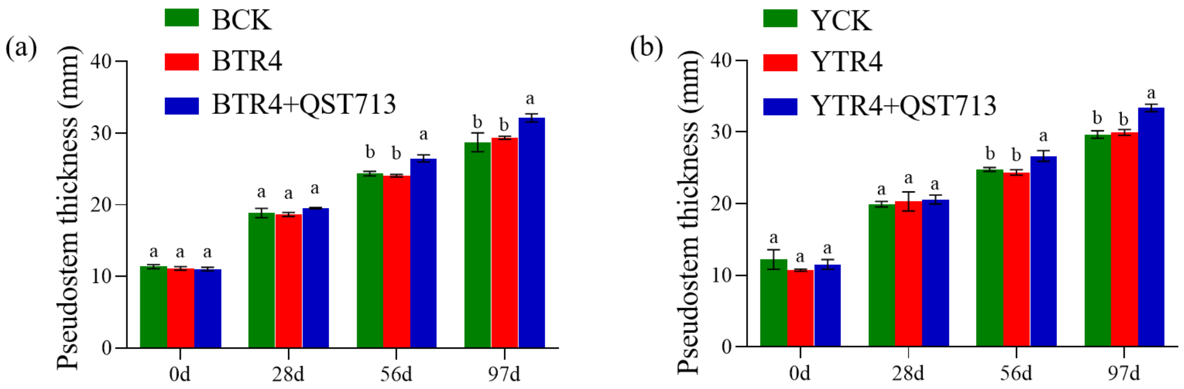

Supplementary Figure S1. Growth-promoting effects of QST713 on banana pseudostem thickness. (a) The pseudostem diameter of Brazilian. (b) The pseudostem diameter of Yunjiao No.1. Data are expressed as mean ± standard error. Data for different lowercase letters indicate a significant difference at the 0.05 level.

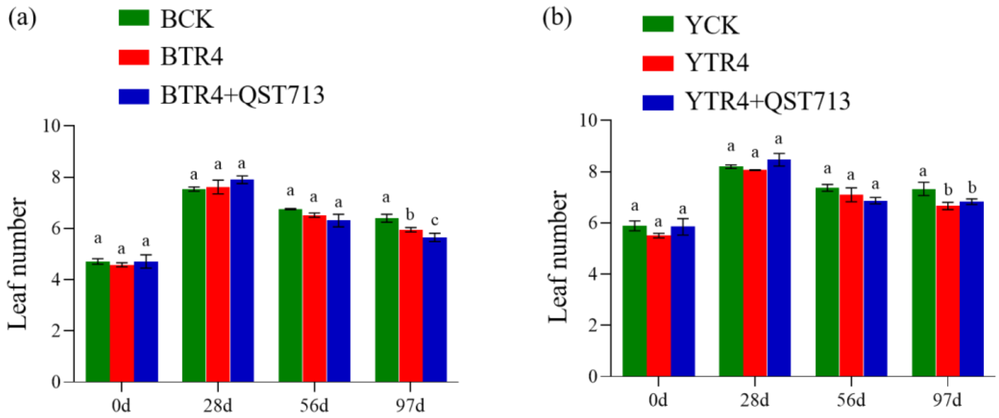

Supplementary Figure S2. Growth-promoting effects of QST713 on banana leaf number. (a) The leaf number of Brazilian. (b) The leaf number of Yunjiao No.1. Data are expressed as mean ± standard error. Data for different lowercase letters indicate a significant difference at the 0.05 level.

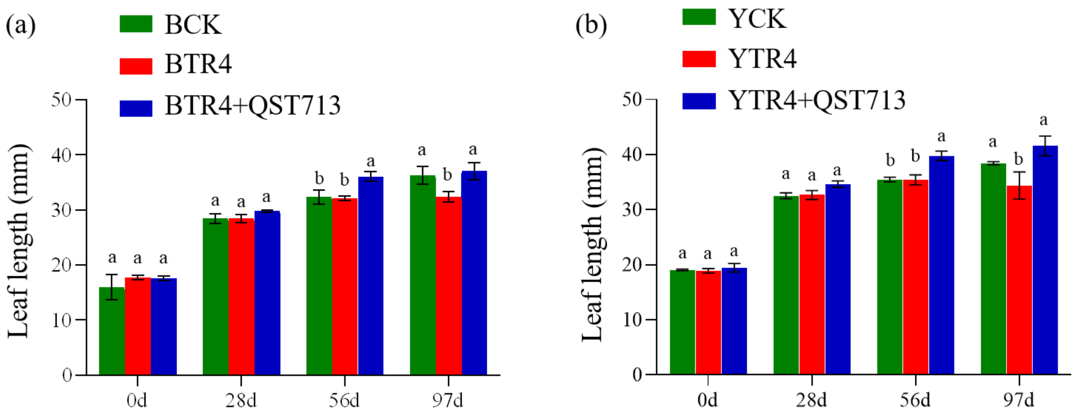

Supplementary Figure S3. Growth-promoting effects of QST713 on banana leaf length. (a) The leaf length of Brazilian. (b) The leaf length of Yunjiao No.1. Data are expressed as mean ± standard error. Data for different lowercase letters indicate a significant difference at the 0.05 level.

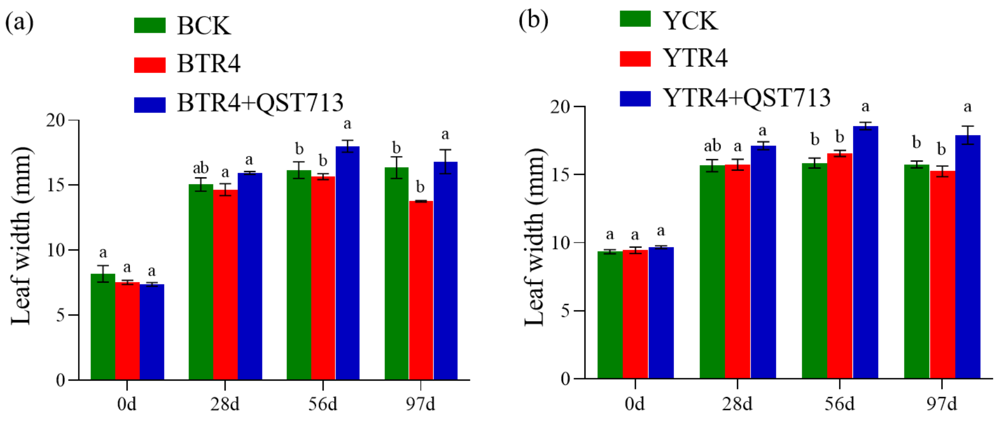

Supplementary Figure S4. Growth-promoting effects of QST713 on banana leaf width. (a)The leaf width of Brazilian. (b) The leaf width of D Yunjiao No.1. Data are expressed as mean ± standard error. Data for different lowercase letters indicate a significant difference at the 0.05 level.

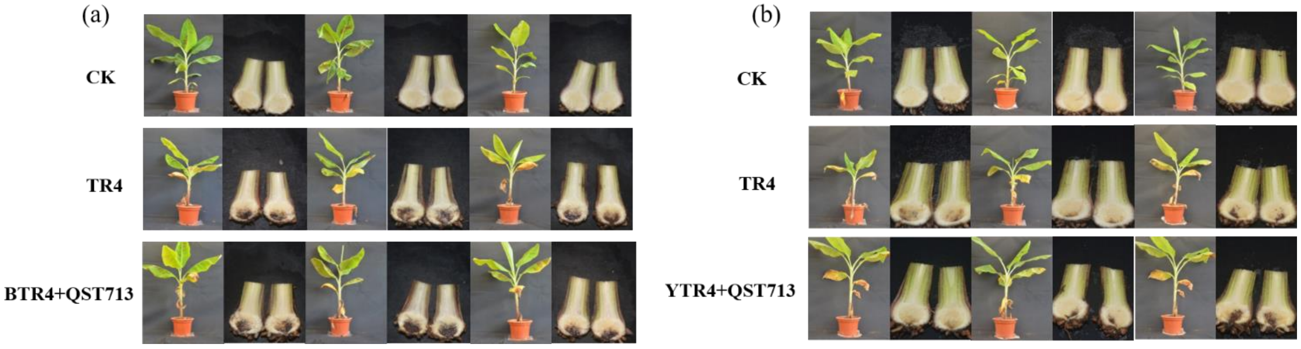

Supplementary Figure S5. Biocontrol effect of QST713 on bananas. (a) Representative images of Brazilian in different treatments. (b) Representative images of Yunjiao No.1 in different treatments

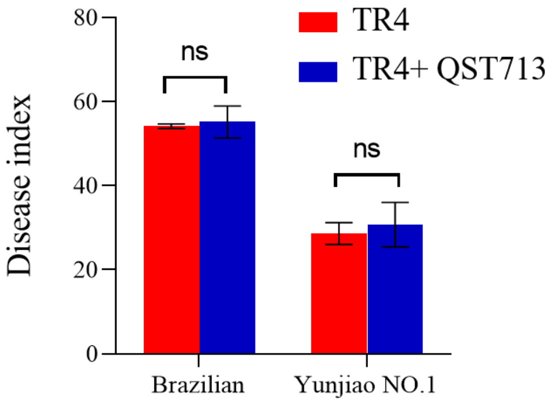

Supplementary Figure S6. Disease index of banana in different treatments.

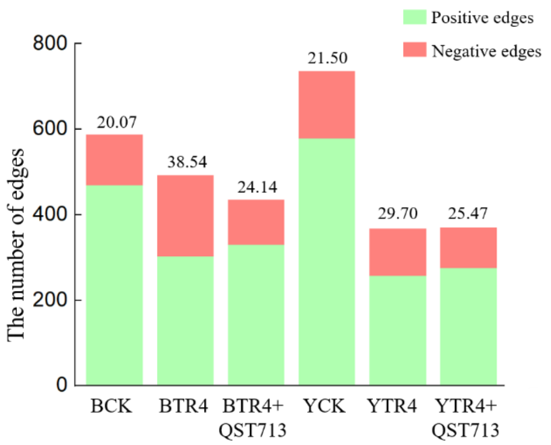

Supplementary Figure S7. Number of negative(green) and positive (red) correlation edges of each network. Proportion (%) of negative correlation edges in the total edges on the histogram.

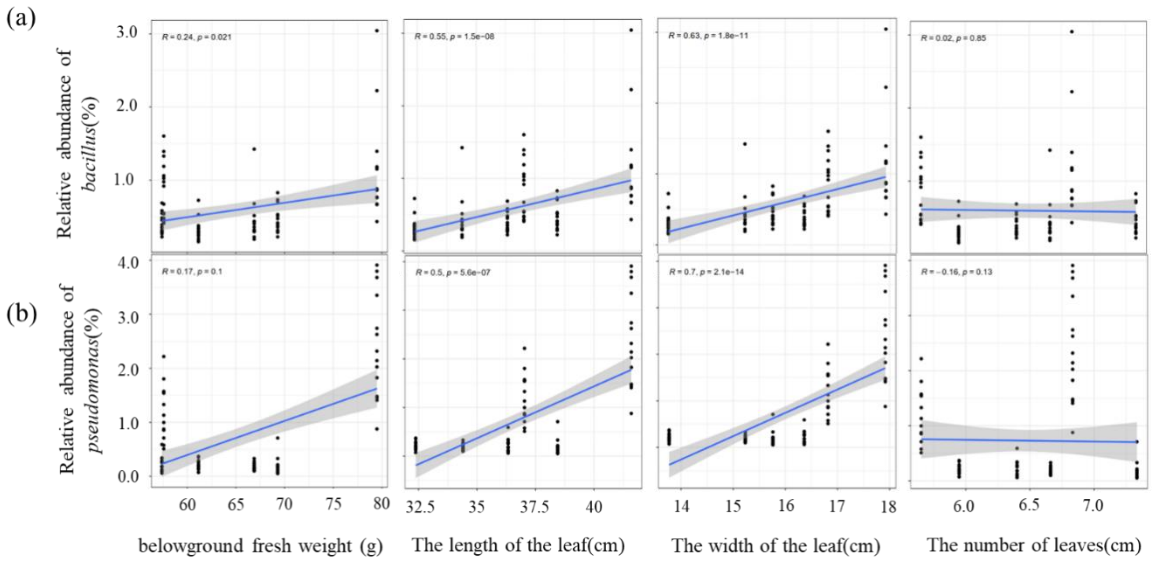

Supplementary Figure S8. Correlation between dominant bacteria and growth traits of banana based on spearman. The y axis indicates the relative abundance of dominant bacteria and the x axis indicates the agronomic traits. Blue lines show linear regression. R represents differential explanation rate, *P* represents significance.
