# Supplementary material for: A biological product of Bacillus amyloliquefaciens QST713 strain for promoting banana plant growth and modifying rhizosphere soil microbial diversity and community composition
Source: Front Microbiol. 2023 Nov 2;14:1216018. doi: 10.3389/fmicb.2023.1216018 (PMC10653307; doi:10.3389/fmicb.2023.1216018)
Supplement: Supplementary file 2 [file Presentation_2.PPTX]

## Slide 1
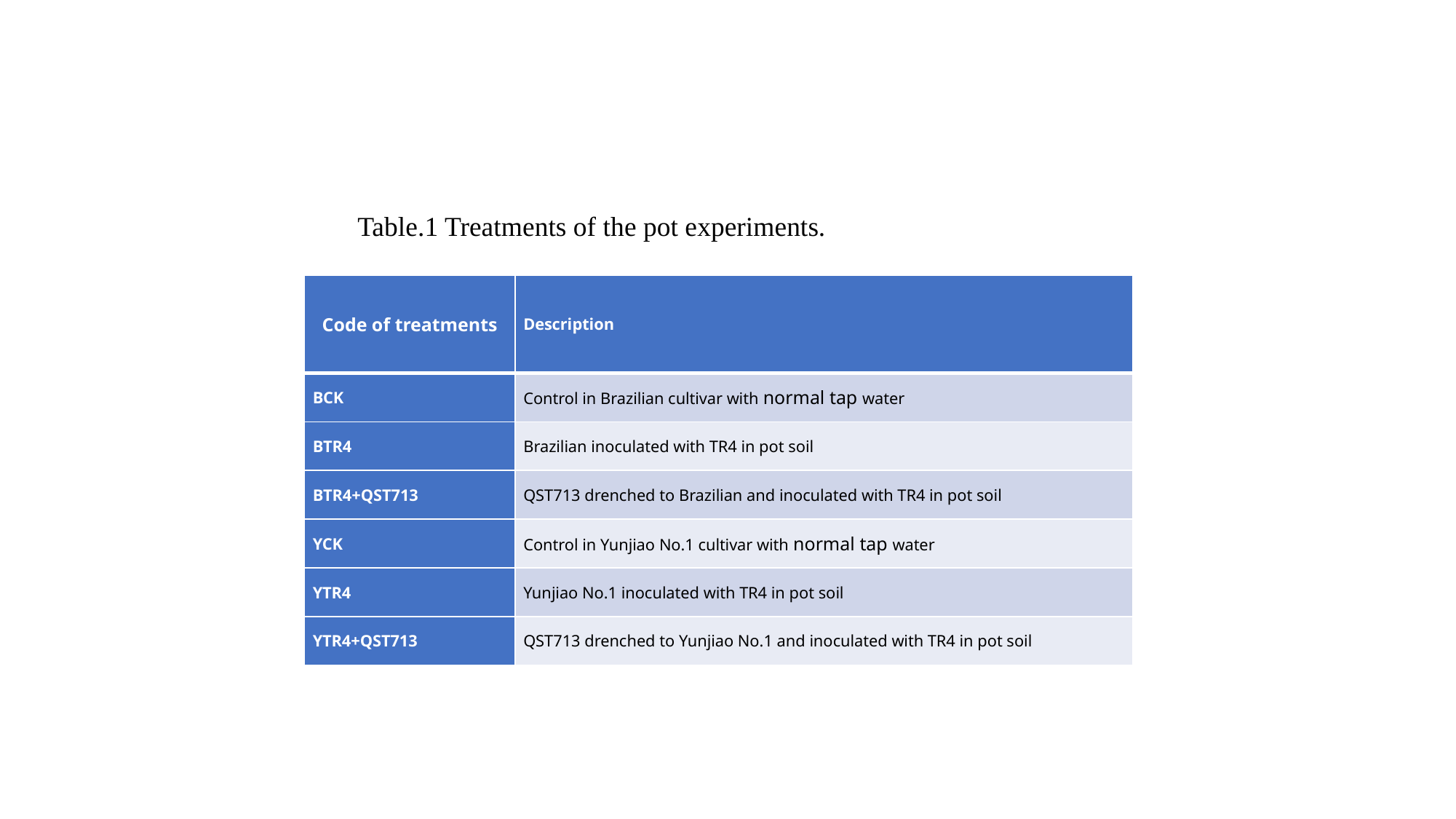

Table.1 Treatments of the pot experiments.
| Code of treatments | Description |
| --- | --- |
| BCK | Control in Brazilian cultivar with normal tap water |
| BTR4 | Brazilian inoculated with TR4 in pot soil |
| BTR4+QST713 | QST713 drenched to Brazilian and inoculated with TR4 in pot soil |
| YCK | Control in Yunjiao No.1 cultivar with normal tap water |
| YTR4 | Yunjiao No.1 inoculated with TR4 in pot soil |
| YTR4+QST713 | QST713 drenched to Yunjiao No.1 and inoculated with TR4 in pot soil |
