# Supplementary figures and images for: A biological product of Bacillus amyloliquefaciens QST713 strain for promoting banana plant growth and modifying rhizosphere soil microbial diversity and community composition
Source: Front Microbiol. 2023 Nov 2;14:1216018. doi: 10.3389/fmicb.2023.1216018 (PMC10653307; doi:10.3389/fmicb.2023.1216018)

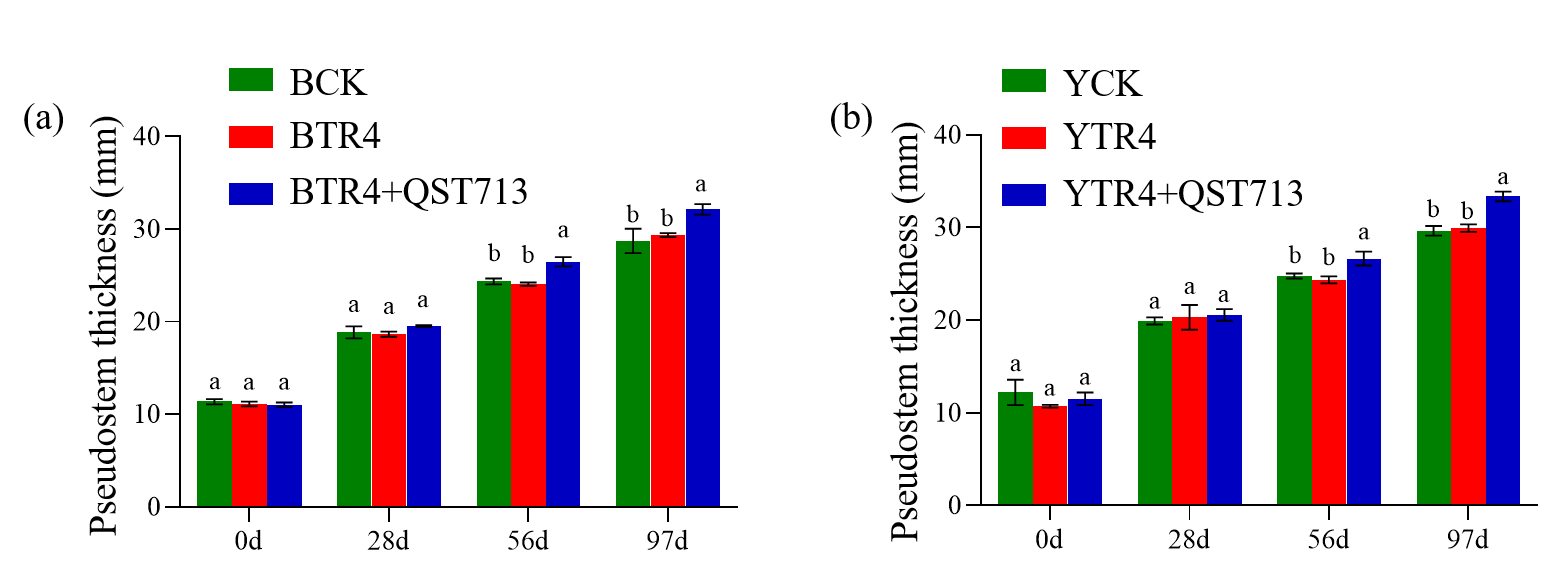

Supplement: Supplementary file 3 [file Image_1.TIF]

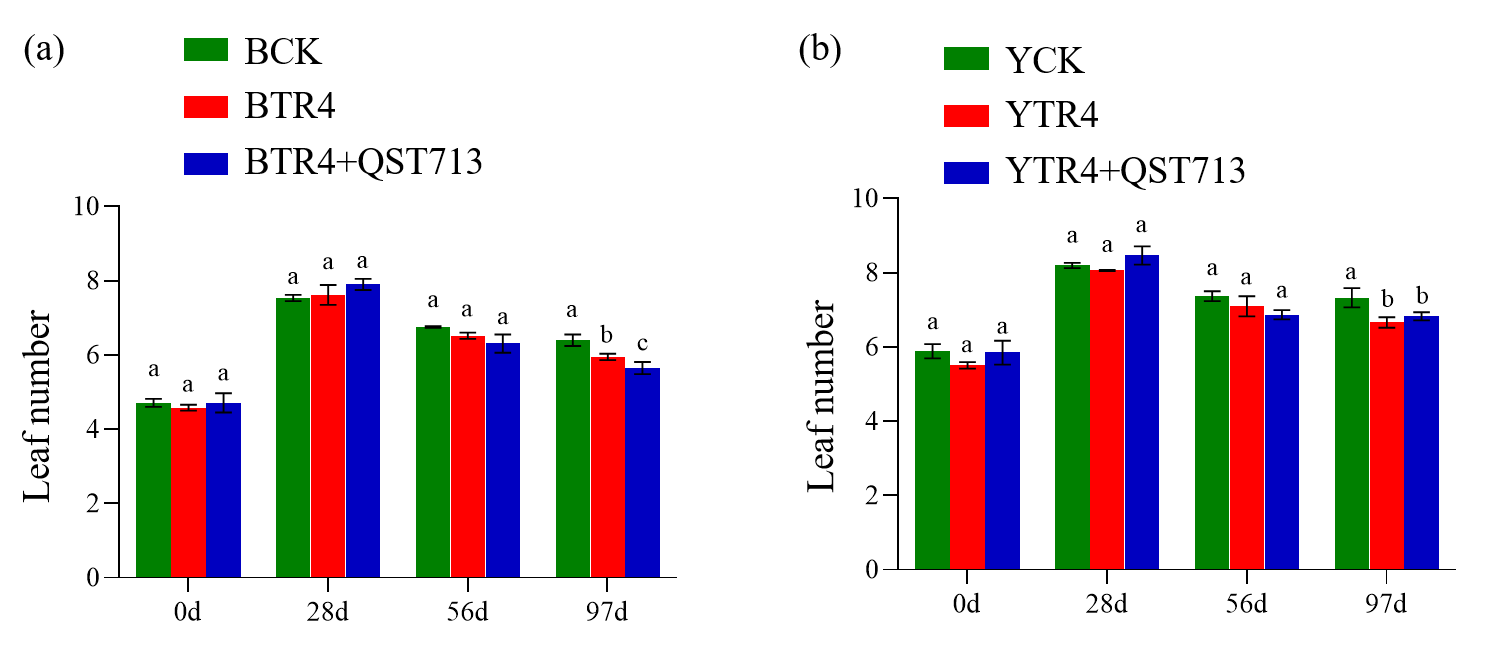

Supplement: Supplementary file 4 [file Image_2.TIF]

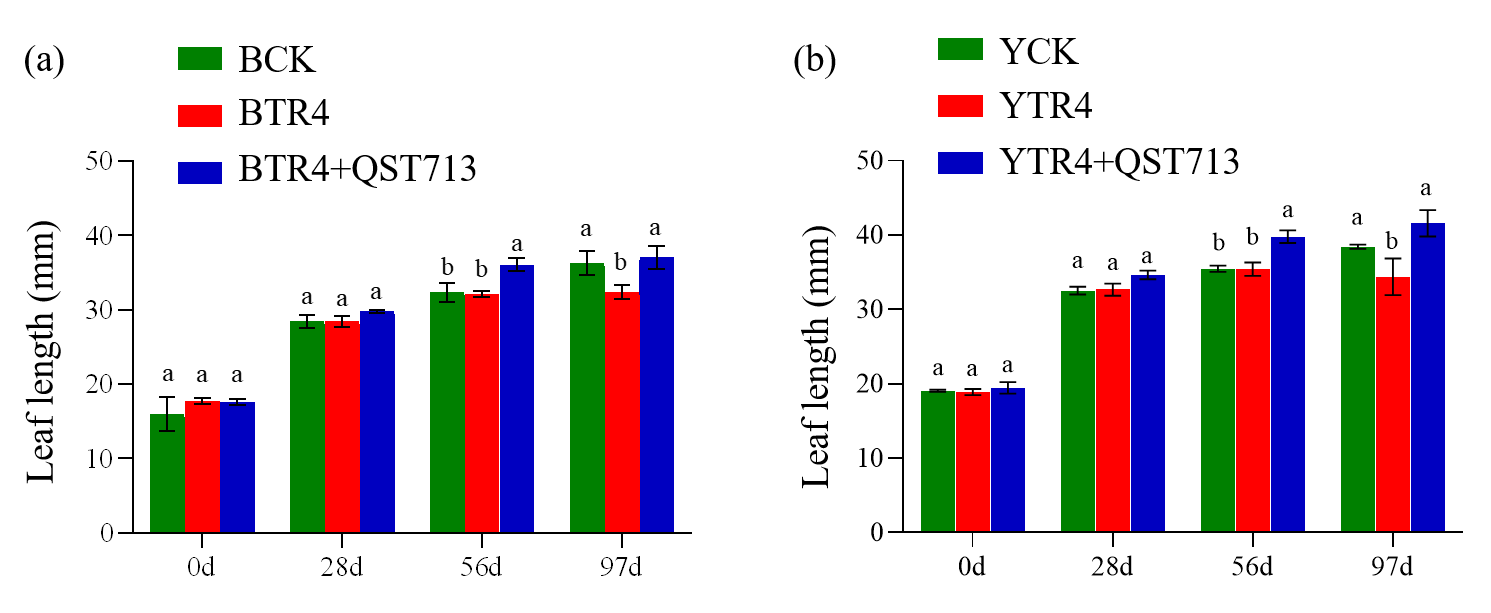

Supplement: Supplementary file 5 [file Image_3.TIF]

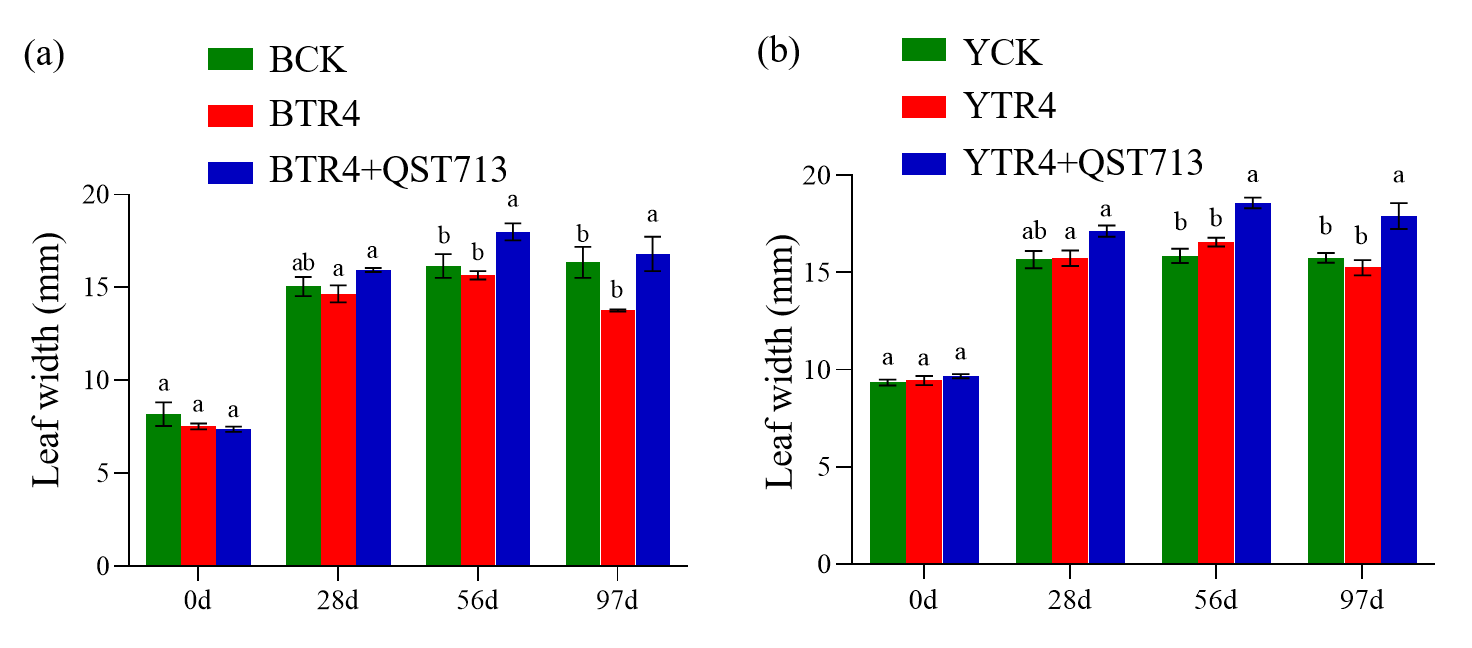

Supplement: Supplementary file 6 [file Image_4.TIF]

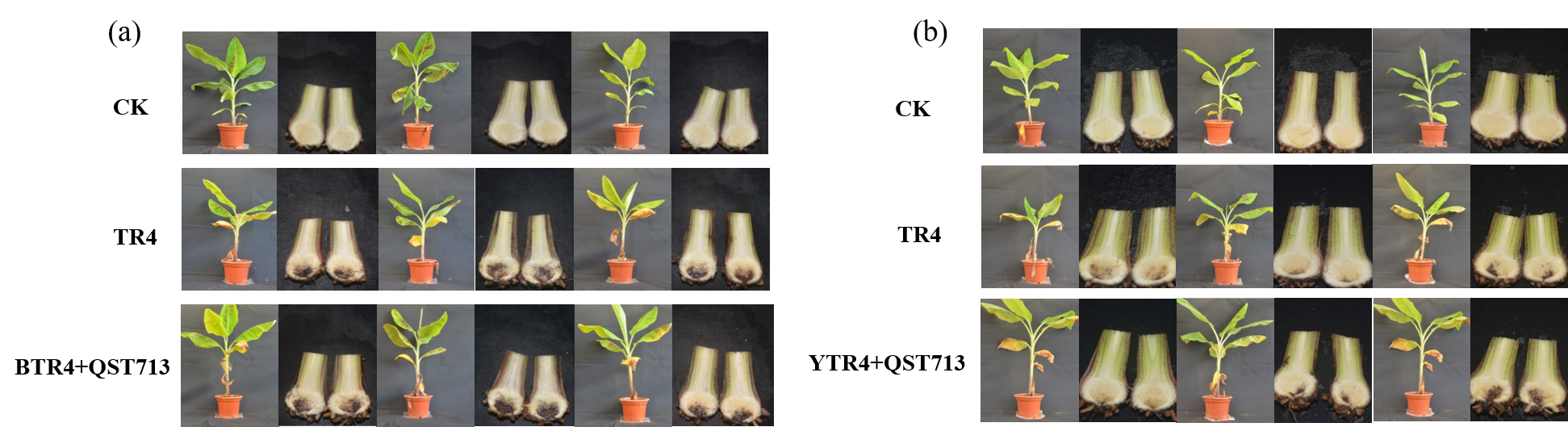

Supplement: Supplementary file 7 [file Image_5.TIF]

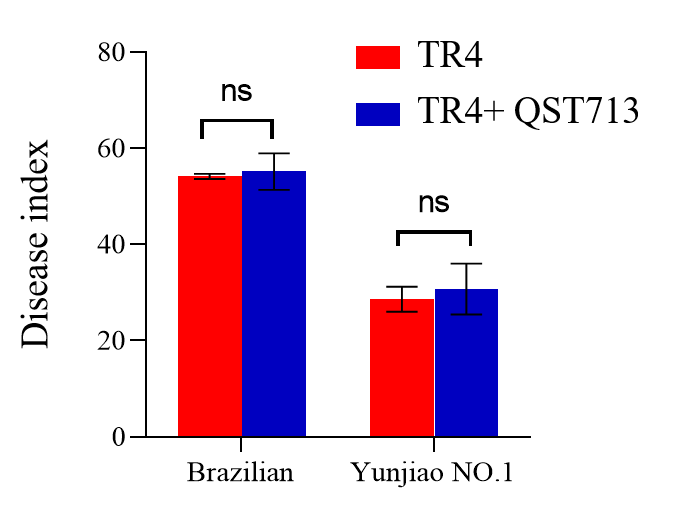

Supplement: Supplementary file 8 [file Image_6.TIF]

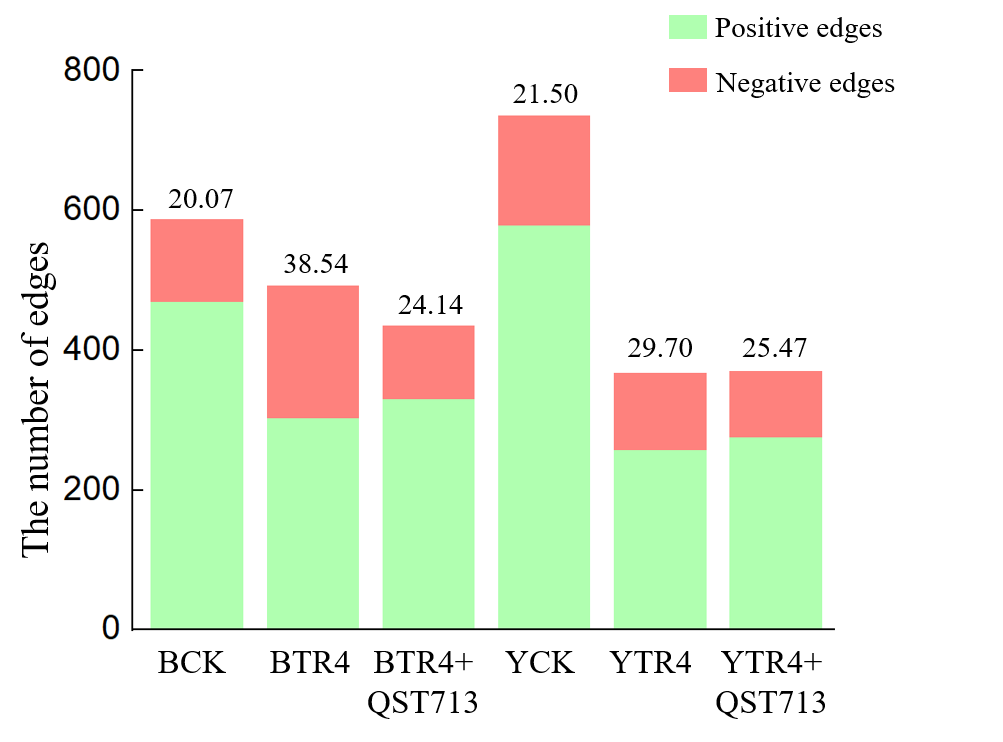

Supplement: Supplementary file 9 [file Image_7.TIF]

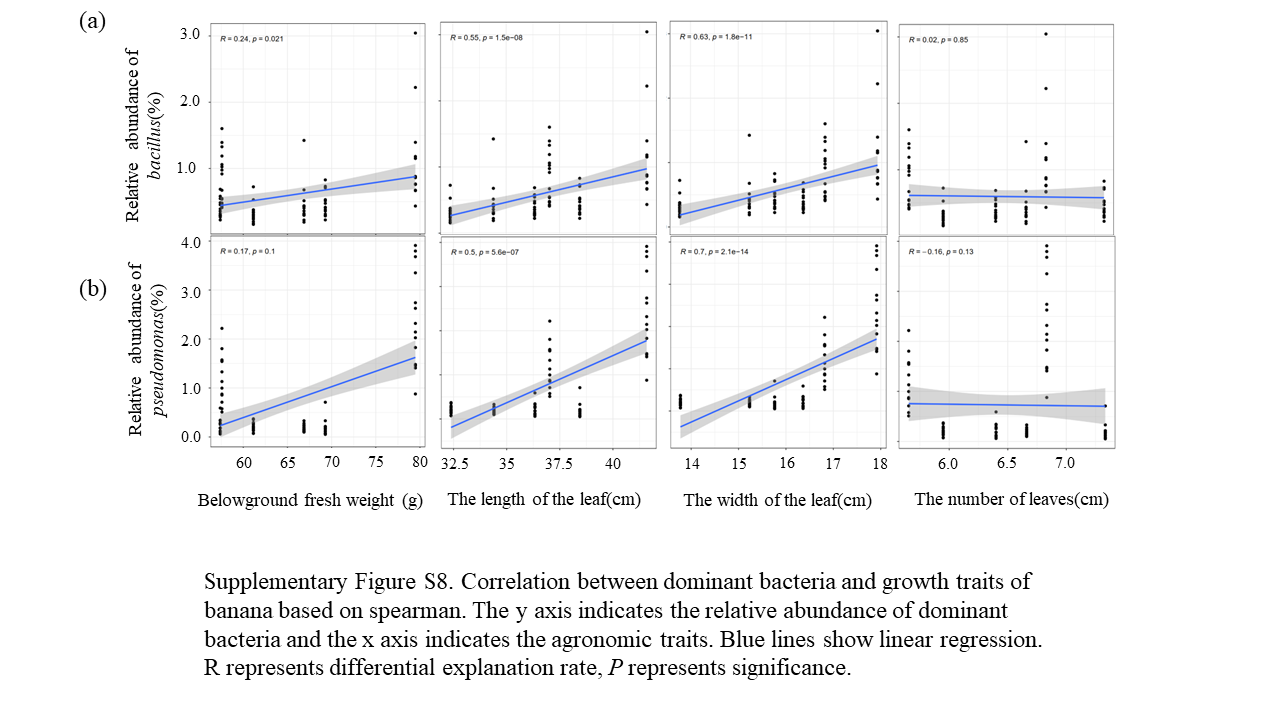

Supplement: Supplementary file 10 [file Image_8.TIF]
